# Serial macromolecular crystallography at ALBA Synchrotron Light Source

**DOI:** 10.1107/S1600577522002508

**Published:** 2022-04-04

**Authors:** Jose M. Martin-Garcia, Sabine Botha, Hao Hu, Rebecca Jernigan, Albert Castellví, Stella Lisova, Fernando Gil, Barbara Calisto, Isidro Crespo, Shatabdi Roy-Chowdhury, Alice Grieco, Gihan Ketawala, Uwe Weierstall, John Spence, Petra Fromme, Nadia Zatsepin, Dirk Roeland Boer, Xavi Carpena

**Affiliations:** a Center for Applied Structural Discovery, Biodesign Institute, Arizona State University, Tempe, AZ, USA; b Department of Crystallography and Structural Biology, Institute of Physical Chemistry Rocasolano, Spanish National Research Council (CSIC), Madrid, Spain; c Department of Physics, Arizona State University, Tempe, AZ, USA; d Molecular Biology Institute of Barcelona, CSIC, Barcelona, Spain; e ALBA Synchrotron, Cerdanyola del Vallès, Barcelona, Spain; f ARC Centre of Excellence in Advance Molecular Physics, La Trobe Institute for Molecular ScienceImaging, Department of Chemistry and Physics, La Trobe University, Melbourne, Australia

**Keywords:** serial synchrotron crystallography, viscous jet, LCP, microcrystal, ALBA, XALOC

## Abstract

The increase in successful adaptations of serial crystallography at synchrotron radiation sources continues. To date, the number of serial synchrotron crystallography (SSX) experiments has grown exponentially, with over 40 experiments reported so far. In this work, we report the first SSX experiments with viscous jets conducted at ALBA beamline BL13-XALOC. Small crystals (15–30 µm) of five soluble proteins (lysozyme, proteinase K, phycocyanin, insulin and α-spectrin-SH3 domain) were suspended in lipidic cubic phase (LCP) and delivered to the X-ray beam with a high-viscosity injector developed at Arizona State University. Complete data sets were collected from all proteins and their high-resolution structures determined. The high quality of the diffraction data collected from all five samples, and the lack of specific radiation damage in the structures obtained in this study, confirm that the current capabilities at the beamline enables atomic resolution determination of protein structures from microcrystals as small as 15 µm using viscous jets at room tem­per­ature. Thus, BL13-XALOC can provide a feasible alternative to X-ray free-electron lasers when determining snapshots of macromolecular structures.

## Abstract 2

We report the first SSX experiments with viscous jets conducted at ALBA beamline BL13-XALOC. We confirm that the current capabilities at BL13-XALOC enable atomic resolution determination of protein structures from microcrystals using viscous jets at room tem­per­ature.

## 1. Introduction

There are currently only five X-ray free-electron laser (XFEL) beamlines available worldwide for high-resolution macromolecular crystallography (MX). These scarce resources con­tinue to be in high demand for complex serial femtosecond crystallography (SFX) experiments at XFELs. Meanwhile, the continuous developments in hardware at modern synchrotrons, including faster detectors and brighter beams, have made standard synchrotron beamlines an attractive alternative for performing serial crystallography experiments. Although the vast majority of routine MX experiments are still performed *via* the oscillation method using monochromatic X-ray beams, there has been an increasing trend to use the serial synchrotron crystallography (SSX) approach to solve protein crystal structures from previously intractable crystals of a few micrometres in size. As of February 2022, over 40 SSX experiments were conducted at synchrotrons using exposure times of a few milliseconds (see review article by Martin-Garcia, 2021 ▸). To date, almost every one of the third and fourth-generation synchrotrons have at least one MX beamline that may be used for serial data collection approaches as a routine method for room-tem­per­ature structure determination of proteins and their complexes. The growing recognition of the capabilities of SSX is leading to a few new microfocus beamlines dedicated, partly or fully, to serial crystallography. Some examples of new MX beamlines cur­rently in operation or under construction are the I24 beamline at Diamond Light Source (Oxford, UK) (Horrell *et al.*, 2021 ▸), the ID29 beamline at the ESRF (Grenoble, France, commissioning, https://www.esrf.fr/id29), the MicroMAX beamline at MAX IV (Lund, Sweden, under development, https://www.maxiv.lu.se/accelerators-beamlines/beamlines/micromax/), TREXX at PETRA III (Hamburg, Germany, operational, http://www.embl-hamburg.de/services/mx/P14_EH2/index.html), BL06-XAIRA at ALBA (Barcelona, Spain, under development; Juanhuix *et al.*, 2019 ▸) and MX3 (Merlbourne, Australia, under development, https://www.ansto.gov.au/high-performance-macromolecular-crystallography-beamline). The successful adaptation of SSX at synchrotrons has been possible mostly due to the development of low-consumption sample delivery methods, such as the use of high-viscosity media, fixed targets and hybrid devices (see Table 1 in the review of Martin-Garcia, 2021 ▸). In addition, SSX has demonstrated a reduction in the radiation damage associated with intense beams and has opened up new opportunities for studying irreversible reactions in crystals on a wide range of time scales (Mehrabi *et al.*, 2019 ▸, 2020 ▸; Schulz *et al.*, 2018 ▸).

The MX beamline at ALBA synchrotron light source, BL13-XALOC, opened for general user operation in July 2012 (Juanhuix *et al.*, 2014 ▸). The beamline delivers a flux of 2 × 10^12^ photons s^−1^ into a variable focus of 50–300 µm (H) × 6–100 µm (V) at a 250 mA storage ring current, with photon energies between 4.6 and 23 keV (2.6–0.52 Å). With its current flexible design, high brightness and mid-size beam capacity, it is focused on solving difficult crystallographic challenges. It supports a wide range of MX data collection methods, including raster optimization of diffraction from inhomogeneous crystals, high-resolution data collection from large-unit-cell crystals, room-tem­per­ature data collection on crystals that are difficult to freeze and the study of conformational dynamics, semi-automated data col­lection for sample-screening and ligand binding studies. We have now augmented its capabilities to include SSX experiments. BL13-XALOC is equipped with a PILATUS 6M photon-counting detector (DECTRIS, Baden, Switzerland), a fast read-out detector that can reduce the data collection time, which is important for applications such as serial crystallography. With associated sample lifetimes as short as a few milliseconds, a new rapid sample-delivery method has been implemented at BL13-XALOC, *i.e.* a high-viscosity injector, which was recently acquired from Uwe Weierstall at Arizona State University (Weierstall *et al.*, 2014 ▸).

In the work presented here, the first SSX experiments with viscous jets at BL13-XALOC have been conducted which have paved the way to obtain the first SSX membrane protein structure at the beamline (Kovalev *et al.*, 2020 ▸). The diffraction data quality from all samples tested in this study confirms that the current capabilities of BL13-XALOC enables high-resolution determination of protein structures from microcrystals as small as 15 µm using viscous jets. In addition, we have analyzed the site-specific radiation damage of the crystals by measuring the *B*-factor of cysteine di­sulfide bonds in two of the proteins analyzed, following an adapted protocol described by Weik *et al.* (2000 ▸), which shows that the approach used in this article has, overall, negligible radiation damage and a negligible effect on cysteine di­sulfides. Thus, BL13-XALOC can provide an interesting alternative to conventional XFELs when determining the static structures of macromolecules. The adaptation of SSX with viscous jets at BL13-XALOC will push the frontier of synchrotron crystallography by enabling its users to determine high-quality structures from difficult-to-crystallize targets like membrane proteins, using crystals of a few micrometres in size.

## 2. Materials and methods

### 2.1. Microcrystal sample preparation

**Figure 1:**
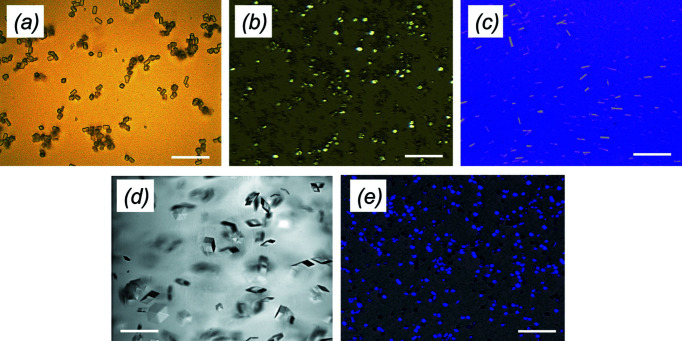
Protein microcrystals at ALBA, showing (*a*) lysozyme, (*b*) proteinase K, (*c*) α-spectrin-SH3, (*d*) insulin and (*e*) phycocyanin. The scale bar represents 100 µm.

Hen egg-white lysozyme and proteinase K from *Tritirachium album* were purchased from Sigma–Aldrich with reference numbers 62970 and P2308, respectively, and their crystals grown on-site in 0.1 *M* sodium acetate (pH 3.0), 18% (*w*/*v*) sodium chloride and 6% PEG 400 for lysozyme, and in 0.1 *M* MES (pH 6.5), 0.5 *M* sodium citrate and 0.1 *M* calcium chloride for proteinase K, using the batch method described previously (Martin-Garcia *et al.*, 2017 ▸). Phycocyanin was puri­fied and microcrystals grown in 75 m*M* HEPES (pH 7.0), 20 m*M* magnesium chloride and 9% PEG 3350 as reported previously (Martin-Garcia *et al.*, 2017 ▸) at Arizona State University labs, and shipped to ALBA experimental laboratories in a 4°C thermo-box prior to the experiment. The SH3 domain of α-spectrin (α-spectrin-SH3) was purified in ALBA experimental laboratories as described previously (Sadqi *et al.*, 1999 ▸) and was subjected to a final purification step using a SEC column previously equilibrated in 10 m*M* citric acid (pH 3) and 150 m*M* sodium chloride. α-Spectrin-SH3 crystals were obtained by applying several concentration/dilution steps with milli-Q water inside standard Amicon Ultra 0.5 ml centrifugal filters (3 kDa cutoff) at 4°C. A crystalline slurry appeared when most of the citric acid buffer was removed. Human insulin was purchased from Sigma–Aldrich with reference number 11376497001. Insulin powder was dissolved in 5 m*M* zinc chloride and 25 m*M* HCl at a concentration of 5–10 mg ml^−1^. Cuboid-shaped microcrystals were obtained in 35.2 m*M* sodium citrate (pH 7) and 5% (*v*/*v*) acetone as precipitant by the batch method using agarose gel at 0.1% as medium for growing the crystals as described previously (Artusio *et al.*, 2020 ▸). Crystal size varied between 20 µm × 10 µm × 5 µm for lysozyme, 15 µm × 10 µm × 5 µm for proteinase K, 20 µm × 15 µm × 5 µm for phycocyanin, 30 µm × 5 µm × 5 µm µm for α-spectrin-SH3 and 30 µm × 30 µm × 30 µm for insulin (Table 1 ▸). Microcrystals of all proteins tested are shown in Fig. 1 ▸. Lipidic cubic phase (LCP) was prepared using 9.9 MAG monoolein (1-oleoyl-*rac*-gly­cerol) purchased from Sigma–Aldrich (M7765) as described previously (https://cherezov.usc.edu/reconstitution.htm). For all samples tested, the crystal density was adjusted before mixing them with LCP, so that mainly single-crystal hits were observed for all samples tested. Crystal mixtures were loaded directly from a Hamilton syringe into the high-viscosity injector (Weierstall *et al.*, 2014 ▸).

### 2.2. Data collection

Diffraction data collection of serial snapshots (*i.e.* no crystal rotation during X-ray exposure) from lysozyme, proteinase K, phycocyanin, α-spectrin-SH3 and insulin microcrystals was performed on the BL13-XALOC end-station at ALBA Synchrotron in Spain (Juanhuix *et al.*, 2014 ▸). Crystals embedded in LCP were streamed across the X-ray beam using a high-viscosity injector (Weierstall *et al.*, 2014 ▸) using a fused-silica capillary with an inner diameter of 50 µm. A 20 µl sample reservoir was used in all experiments. Measurements were performed using the experimental set-up described in §3.1. The crystal carrier stream was extruded out of the nozzle by a pressure that varied between 0.1 and 2.1 MPa, depending on the flow rate of the sample, which was varied from 29.4 to 88.2 nl min^−1^, depending on the sample nature (buffer composition and crystal density) and the observed diffraction, corresponding to an average jet velocity of between 249.6 and 749 µm s^−1^ in the 50 µm diameter nozzle. To prevent the viscous medium stream from curling, a helium-gas sheath was introduced at the point of extrusion. An in-line high-resolution microscope was used to align the nozzle to the beam and to observe the stream. Data were collected at an X-ray energy of 12.6 keV (0.98 Å) for all proteins except for insulin, where the data set was collected at the Zn *K*-edge of 9.7 keV (1.28 Å). All snapshots for all proteins were collected on a PILATUS 6M detector running in a continuous shutterless mode at a frame rate of 12 Hz (80 ms exposure time), while crystals passed through an X-ray beam of 50 µm × 10 µm (H × V) in size. Since crystals had similar diffraction quality, the sample-to-detector distance was kept constant at 506 mm. Data collection statistics are shown in Table 1 ▸.

### 2.3. Data processing and structure determination

All images (in cbf format) were subjected to peak finding, indexing and merging using the software package *CrystFEL* (White, 2019 ▸; White *et al.*, 2012 ▸). For all proteins, all steps of this analysis were performed using *CrystFEL* Version 0.10.0 (https://www.desy.de/~twhite/crystfel/download.html). The peak­finder8 algorithm was used for peak finding with the following parameters: threshold = 30; min-snr = 2; int-rad = 2, 4 and 6; and indexing was performed using *XGANDALF* (Gevorkov *et al.*, 2019 ▸), *MOSFLM* (Powell *et al.*, 2013 ▸) and *DirAx* (Duisenberg, 1992 ▸), in that order.

Lysozyme and proteinase K were solved in the space group *P*4_3_2_1_2 using a tetragonal cell (*a* = *b* = 79.0, *c* = 38.2 Å and α = β = γ = 90° for lysozyme, and *a* = *b* = 68.4, *c* = 108.4 Å and α = β = γ = 90° for proteinase K), and merged in point the group 4/*mmm*. α-Spectrin-SH3 was indexed and merged in the space group *P*2_1_2_1_2_1_ using the orthorhombic cell (*a* = 34.2, *b* = 42.6, *c* = 50.8 Å and α = β = γ = 90°) and merged in the point group *mmm* using *partialator* by applying three iterations and the ‘unity’ model (White, 2019 ▸). For all proteins, the merged *hkl* files were converted to the *mtz* format with the intermediate step of first converting to *XSCALE* format and then to *mtz* using the *XDS* program (Kabsch, 2010 ▸). The intensities were converted to structure factor amplitudes using *AIMLESS* (Evans, 2011 ▸; Evans & Murshudov, 2013 ▸) from the CCP4 program suite (Winn *et al.*, 2011 ▸). While all of the collected data were included for structure determination (see Tables S1 and S2 of the supporting information), it can be seen that ∼50 000 indexable patterns for proteinase K and ∼10 000 indexable patterns for lysozyme would be sufficient for reaching acceptable data-quality statistics and full completeness for the same resolution range. This would correspond to data collection times of only ∼2 and 1 h, respectively.

The lysozyme and proteinase K phases were calculated by molecular replacement with *MOLREP* (Lebedev *et al.*, 2008 ▸; Vagin & Teplyakov, 2010 ▸) using the PDB entries 1vds (un­published work) and 1ic6 (Betzel *et al.*, 2001 ▸), respectively, as reference models, with all solvent atoms removed. α-Spectrin-SH3 phases were determined with *MOLREP* using PDB entry 4f17 (unpublished work) as the search model, with all solvent atoms removed. Insulin was solved in the space group *H*3 (unit-cell dimensions *a* = *b* = 81.6, *c* = 33.6 Å, α = β = 90° and γ = 120°) of a trigonal cell with hexagonal axes. After in­dexing, *ambigator* was used to resolve the indexing ambiguity due to serial data collection for this point group (applying operators *k*, *h*, −*l*; −*h*, *h*+*k*, −*l* and −*h*, −*k*, *l*) and then merged into point group *-3_H*. Phycocyanin was indexed into the hexagonal unit cell *a* = *b* = 188.5, *c* = 61.0 Å, α = β = 90 and γ = 120°, and the indexing ambiguity solved prior to merging into *-3 m1_H*. Phasing was performed with *MOLREP* using the coordinates from PDB entry 5uvk (Martin-Garcia *et al.*, 2017 ▸) for phycocyanin and 4ey9 (Fávero-Retto *et al.*, 2013 ▸) for insulin as reference models. All five structures were refined using alternate cycles of restrained refinement with *REFMAC5* (Murshudov *et al.*, 2011 ▸) and manual refinement in *COOT* (Emsley *et al.*, 2010 ▸). The structures were then submitted to the PDB-REDO server (Joosten *et al.*, 2009 ▸) for final refinement before being de­posited in the PDB.

The maximum radiation dose per crystal was estimated using the RADDOSE-3D server (Zeldin *et al.*, 2013 ▸), assuming cuboid crystals for all four crystal dimensions tested in this study (see §2.2), a beam size of 50 µm × 10 µm, a photon flux of 2 × 10^12^ photons s^−1^, an energy of 12.6 keV and an exposure time of 80 ms. These estimates varied from 98 to 150 K Gy per crystal, as summarized in Table 1 ▸. Crystal structure superposition and calculation of the root-mean-square deviation (RMSD) values were performed using the *LSQKAB* program (Kabsch, 1976 ▸). All figures were prepared with *UCSF CHIMERAX* (Version 1.2.4) (Goddard *et al.*, 2018 ▸; Pettersen *et al.*, 2004 ▸).

### 2.4. Site-specific radiation damage analysis

Site-specific radiation damage analysis was carried out on all protein structures presented in this study using the *RABDAM* program (Shelley *et al.*, 2018 ▸), recently implemented in the CCP4 program suite (Winn *et al.*, 2011 ▸). An adaptation of the available Python scripts reported by Shelley *et al.* was used to obtain the data files from the analyzed PDBs and to draw the plots showing the *B*_Damage_ distributions corresponding to either the whole protein, some side-chain terminal atoms or cysteine S atoms (always excluding all H atoms). We applied default values of 7 Å to calculate the packing density and a sliding window of 2%, as suggested in Shelley *et al.* (2018 ▸).

The site-specific radiation damage was further evaluated by recording the *B*-factor of the final refined models of the atoms of the cysteine residues of lysozyme and proteinase K involved in di­sulfide bonds. The residue pairs in question were Cys6/Cys127, Cys30/Cys115, Cys64/Cys80 and Cys76/Cys94 for lysozyme, and Cys34/Cys123 and Cys178/Cys249 for proteinase K. The ratios between the S_γ_ and C_β_ atoms were calculated for all the individual residues, and then these ratio values were averaged for the S_γ_/C_β_ pairs for each respective di­sulfide bond, resulting in a single number for each cysteine pair forming a di­sulfide bond. For comparison, these numbers were also calculated for a series of relevant PDB structures as described in the *Results and discussion* section (§3).

## 3. Results and discussion

**Figure 2:**
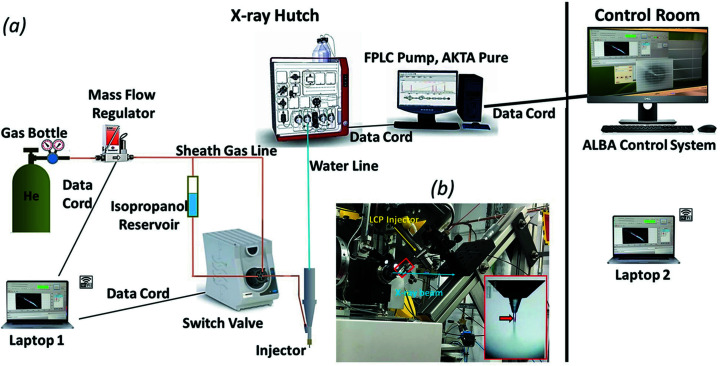
Experimental 45° set-up at BL13-XALOC. (*a*) Schematic diagram of the high-viscosity injector operation system showing all equipment and parts used for the set-up. A conventional FPLC pump was used. Helium was used as sheath gas in all our experiments. (*b*) High-viscosity injector (Weierstall *et al.*, 2014 ▸) mounted on translation stages. The inset panel shows a closer view of the LCP stream extruding from a 50 µm glass capillary nozzle. The red arrow indicates the position of the X-ray beam.

In this study, we demonstrate that SSX with viscous jets is feasible at BL13-XALOC and can be used to determine the structures of a range of proteins of different sizes and types from crystal sizes between 15 and 30 µm. To deliver the microcrystals into the X-ray beam, we used a high-viscosity injector (Weierstall *et al.*, 2014 ▸). Diffraction data were collected in a continuous shutterless mode using a PILATUS 6M detector operating at 12 Hz. The experimental set-up is shown in Fig. 2 ▸. We successfully collected excellent quality full data sets for five different proteins, presented below. In addition to the standard model system, *i.e.* proteins such as hen egg-white lysozyme and proteinase K from *Tritirachium album limber*, we included phycocyanin from *Thermosynechococcus elongatus*, human insulin and the SH3 domain of α-spectrin (α-spectrin-SH3) from *Gallus gallus*.

### 3.1. Experimental set-up at the BL13-XALOC end-station

Serial data collection was performed at BL13-XALOC at ALBA, a third-generation synchrotron radiation source (Barce­lona, Spain). A detailed description of the beamline end-station can be found in Juanhuix *et al.* (2014 ▸). BL13-XALOC is a 30 m-long (from the photon source) energy-tunable beamline at the 3 GeV storage ring of ALBA which operates at a final current of 250 mA. It is fed by a 2 m-long in-vacuum undulator (IVU21) (Bruker Advanced Supercon, Bergisch Gladbach, Germany), with a minimum gap of 5.7 mm, and placed in a medium straight section of the storage ring. The focused X-ray beam has a cross-section of 50 µm × 10 µm full width at half-maximum (FWHM) at the sample position, with a photon flux of 2 × 10^12^ photons s^−1^ at 250 mA ring current and an energy ranging from 4.6 to 23 keV. The beamline optics consist of an Si(111) double-crystal monochromator and a pair of focusing mirrors in Kirkpatrick–Baez geometry (Kirkpatrick & Baez, 1948 ▸).

The BL13-XALOC end-station is based on two in-house-developed positioning tables (Colldelram *et al.*, 2010 ▸) that support the detector and the diffractometer together with the beam-conditioning elements. The sample-viewing system, an OAV B-ZOOM (ARINAX), consists of an on-axis parallax-free high-resolution video microscope (0.16 µm/pixel at maximum zoom) and a user-controlled front light and a polarized back light (transmitted). The experimental station consists of an MD2M diffractometer (Maatel), a PILATUS 6M hybrid pixel detector (DECTRIS, Switzerland), which can collect full-frame data with a frequency of up to 12.5 Hz in shutterless mode, and a CATS automatic sample exchanger (IRELEC).

To avoid collision with existing hardware, the sample injector was installed on a fixed translational stage at 45° to the X-ray beam. A schematic view of the experimental set-up used at BL13-XALOC is shown in Fig. 2 ▸. A standard fast protein liquid chromatography (FPLC) instrument (ÄKTA Pure 25M) was used to extrude the samples out of the injector, and helium was employed as sheath gas to keep the extruded stream stable. For the experiment, an upgraded version of the injection system used in previous experiments at synchrotrons (Martin-Garcia *et al.*, 2017 ▸, 2019 ▸; Nogly *et al.*, 2015 ▸) was installed at BL13-XALOC. The upgrades include the following modifications: (1) the output of a pressurized iso­propanol reservoir can be coupled into the sheath gas line through a Rheodyne valve (IDEX MXP7970-000), which en­abled switching between sheath gas and iso­propanol at the nozzle when needed; (2) a remote-control system for the Rheodyne valve and gas flow controller comprised of two laptops (one in the X-ray hutch and another in the control room), connected wirelessly. This enabled us to remotely control the gas flow and wash the aperture of the nozzle with iso­propanol (to remove viscous medium build-up) from outside the X-ray hutch if the sample stream was not stable or if it clogged. In addition, the new injector reservoirs are made of titanium, which is more durable and resistant to corrosion.

### 3.2. Structural data analysis

#### 3.2.1. Model proteins lysozyme and proteinase K

Both lysozyme and proteinase K were crystallized, pelleted and then suspended in LCP as described previously (Martin-Garcia *et al.*, 2017 ▸). The corresponding microcrystal–LCP suspension containing lysozyme or proteinase K microcrystals were then loaded into the high-viscosity injector (Weierstall *et al.*, 2014 ▸) and diffraction data were collected using a flow rate of 29.4 nl min^−1^ for both samples. Lysozyme and proteinase K crystals belonged to the space group *P*4_3_2_1_2, and data were recorded up to maximum resolutions of 2.1 and 1.9 Å, respectively. For lysozyme, a total of 114080 images were recorded in an effective measuring time of 2 h 34 min, 23733 of which were successfully indexed (combined hit and indexing rate = 21%) and merged. A diffraction pattern from a lysozyme microcrystal is shown in Fig. S1 (see supporting information). The structure of lysozyme was solved by molecular replacement using the previously solved structure (PDB entry 1vds, to be published) as a reference model with all solvent molecules removed. The final structure was refined to 2.1 Å resolution with *R*_work_ and *R*_free_ values of 17.9 and 23.2%, respectively (Table 2 ▸). As for proteinase K, 577904 images were recorded in 13 h 46 min, which resulted in a sample consumption of 19.4 µl. This resulted in a total of 216645 successfully indexed (combined hit and indexing rate = 32%) and merged patterns. Despite carefully mixing of the microcrystals with LCP to reach a homogenous sample, multi-crystal diffraction was observed (Fig. S2 in the supporting information). The *CrystFEL* internal architecture (Version 0.5.0; https://www.desy.de/~twhite/crystfel/relnotes-0.5.0) was changed so that it can index multiple crystal lattices in one image. The structure of proteinase K was solved by molecular replacement using the previously solved cryogenic structure (PDB entry 1ic6; Betzel *et al.*, 2001 ▸) as a reference model with all solvent molecules removed. The final structure was refined to 1.9 Å resolution with *R*_work_ and *R*_free_ values of 16.5 and 19.6%, respectively (Table 2 ▸). The final data collection and refinement statistics of lysozyme and proteinase K are given in Tables 1 ▸ and 2 ▸.

**Figure 3:**
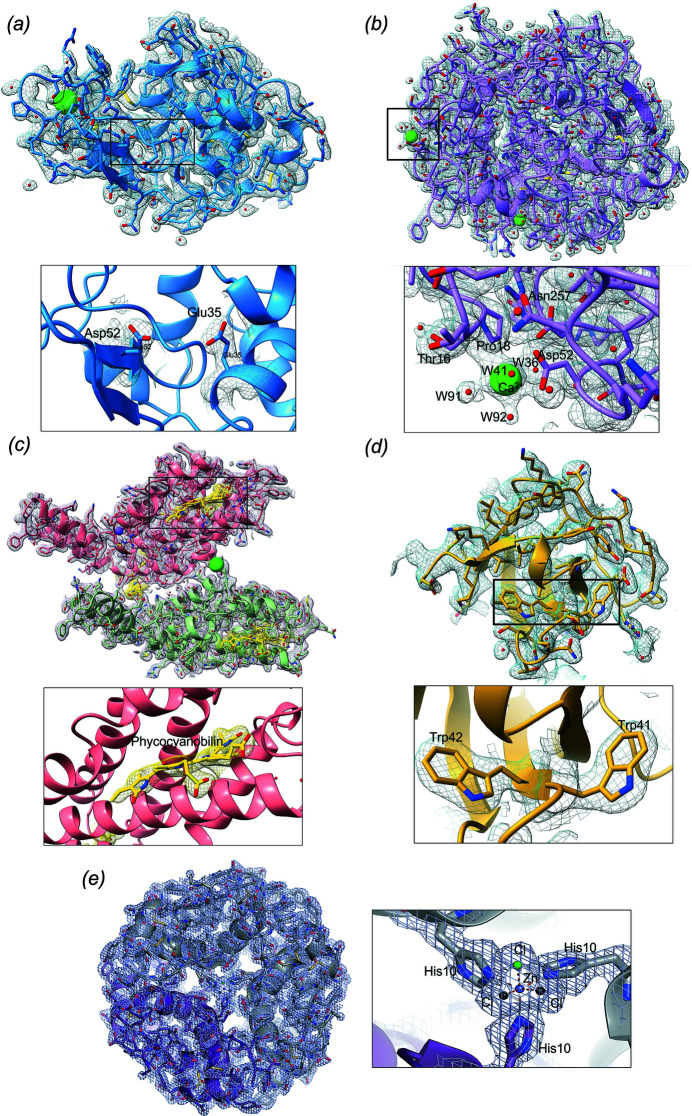
Quality of the 2mFo-DFc electron density maps for all structures tested contoured at 1σ. (*a*) Lysozyme illustrated as a blue cartoon and stick representation. Solvent molecules (water in red) and Cl^−^ ions (green) are represented as spheres. A closer view (panel below) of the Glu35 and Asp52 residues at the catalytic site. (*b*) Proteinase K illustrated as a pink cartoon and stick representation. Solvent molecules (water in red) and the Ca^2+^ ion (green) are represented as spheres. A closer view (panel below) of one of the Ca^2+^ ions. (*c*) The two phycocyanin subunits (α green and β salmon) illustrated as a blue cartoon and stick representation. The chromophores are shown in a yellow cartoon and stick representation. Solvent molecules (water in red), Na^+^ ions (purple) and Cl^−^ ions (green) are represented as spheres. A closer view (panel below) of the phy­co­cyano­bilin chromophore. (*d*) The α-spectrin-SH3 domain illustrated as a yellow cartoon and stick representation. Water molecules are shown as red spheres. A closer view (panel below) of the Trp41 and Trp42 residues at the binding site. (*e*) Hexameric human insulin illustrated as a cartoon and stick representation. The dimer in the asymmetric unit is shown in purple and symmetry-related molecules are shown in dark grey. A closer view (right panel) of the coordination of the Zn^2+^ ion (blue) with the His10 residues (sticks), and the Cl^−^ ions (in green, and black for the symmetry-related molecules).

The high quality of the lysozyme and proteinase K structures can be assessed from the electron density maps 2mFo-DFc for the active site residues of lysozyme, and the two calcium ions, and neighbouring residues of proteinase K (Fig. 3 ▸). Further evaluation of the quality of the structures was carried out by comparing them with previously reported crystal structures determined at room tem­per­ature using SSX, such as PDB entries 4rlm (Botha *et al.*, 2015 ▸), 4z98 (Murray *et al.*, 2015 ▸) and 4x3b (Roedig *et al.*, 2015 ▸) for lysozyme, and 5uvl (Martin-Garcia *et al.*, 2017 ▸), 6fjs (Botha *et al.*, 2018 ▸) and 5mjl (Meents *et al.*, 2017 ▸) for proteinase K. Overall, all the lysozyme and proteinase K structures aligned very well with each other, with average RMSD values of 0.215 and 0.172 Å for the C^α^ atoms of lysozyme and proteinase K, respectively. The average RMSD values for all atoms (0.681 and 0.447 Å for the C^α^ atoms of lysozyme and proteinase K, respectively) indicate slightly higher differences, which are mainly found in the loop regions, as well as in the solvent-exposed regions, as one would expect.

#### 3.2.2. Phycocyanin

Microcrystals of phycocyanin, a photosynthetic pigment from the thermophilic cyano­bac­ter­ium *T. elongatus*, were suspended in LCP and extruded at a flow rate of 29.4 nl min^−1^. A total of 753533 images were collected from 30 µl of sample in about 23 h (Table 1 ▸), from which 152142 patterns were successfully indexed (combined hit and indexing rate = 20%) and merged. A representative diffraction pattern from the phycocyanin sample is shown in Fig. S3 (see supporting information). The structure of phy­co­cyanin was determined by molecular replacement using the previously solved PDB crystal structure 5uvk (Martin-Garcia *et al.*, 2017 ▸) as the search model and refined to a 2.1 Å resolution with final *R*_work_ and *R*_free_ values of 30.5 and 34.7%, respectively (Table 2 ▸). The final data collection and refinement statistics are shown in Tables 1 ▸ and 2 ▸. The quality of our structural model can be assessed from the 2mFo-DFc electron density maps for one of the chromophores of the protein, shown in Fig. 3 ▸.

Further assessment of the quality of the obtained structure was carried out by superimposing it with room-tem­per­ature structures: the two SSX structures recently described using viscous jets, PDB entries 5uvk (Martin-Garcia *et al.*, 2017 ▸) and 5mjq (Meents *et al.*, 2017 ▸), and the two SFX structures determined by Fromme and co-workers, PDB entries 4ziz (Fromme *et al.*, 2015 ▸) and 4q70 (to be published). As indicated by the RMSD values for the C^α^ atoms (0.366 Å) and all atoms (0.480 Å), overall, our structure superimposed very well with all phycocyanin structures, with slight differences observed in the solvent-exposed regions.

#### 3.2.3. α-Spectrin-SH3

The α-spectrin-SH3 domain is a small module of about 60 residues found in the α-spectrin protein that mediate transient protein–protein interactions relative to many cellular processes by recognizing polyproline-rich motifs in its protein targets (Kurochkina & Guha, 2013 ▸). Microcrystals of the α-spectrin-SH3 domain were suspended in LCP and delivered to the X-ray beam at a flow rate of 71.4 nl min^−1^ for about 3 h 39 min to collect a total of 139971 images from nearly 14 µl of sample. From all patterns col­lected, 13039 patterns were successfully indexed (combined hit and indexing rate = 9%) and merged. A diffraction pattern representative of the α-spectrin-SH3 microcrystals sample is shown in Fig. S4 (see supporting information). The structure of the α-spectrin-SH3 domain was determined by molecular replacement using the synchrotron structure at 100 K reported by Cámara-Artigas *et al.* (PDB entry 4f17, to be published), and refined to a 2.1 Å resolution with final *R*_work_ and *R*_free_ values of 19.4 and 23.94%, respectively. The data collection and refinement statistics are summarized in Tables 1 ▸ and 2 ▸.

The quality of the α-spectrin-SH3 structure was further evaluated by comparing it with two previously reported cryo-cooled structures, namely, PDB entries 4f16 (Cámara-Artigas and Gavira, unpublished results) and 2nuz (Agarwal and co-workers, unpublished results). Based on the RMSD values for the C^α^ atoms (0.265 Å) and all atoms (0.762 Å), our structure aligned very well with the three α-spectrin-SH3 structures, with major coordinate differences observed in the loops regions, as reported in previous structures (Cámara-Artigas *et al.*, 2011 ▸).

#### 3.2.4. Insulin

Cuboid-shaped microcrystals of insulin grown in agarose gel at 0.1% (Artusio *et al.*, 2020 ▸) were suspended in LCP and extruded at a flow rate of 71.4 nl min^−1^ for 10 h 28 min to collect a total of 433 986 images, with an effective consumption of 30 µl of sample suspension. A total of 51144 patterns were successfully indexed (combined hit and indexing rate = 12%) and merged. A diffraction pattern representative of a single insulin microcrystal is shown in Fig. S5 (see supporting information). All data collection statistics are shown in Table 1 ▸. The structure of insulin was determined by molecular replacement using the crystal structure for PDB entry 4ey9 (Fávero-Retto *et al.*, 2013 ▸) as the search model. The structure, containing two protein monomers and three ions (2 × Zn^2+^ plus 1 × Cl^−^) in the asymmetric unit forming the typical T6 hexameric assembly of human insulin, was refined to 1.7 Å, with final *R*_work_ and *R*_free_ values of 24.0 and 25.8%, respectively (Table 2 ▸). The quality of our structural model can be assessed from the 2mFo-DFc electron density maps for one of the two Zn^2+^ ions (Fig. 3 ▸).

To evaluate the quality of our structure model, we superimposed it with previously reported cryogenic human insulin structures, PDB entries 1os3 (Smith & Blessing, 2003 ▸), 4f0n (Fávero-Retto *et al.*, 2013 ▸) and 5co2 (unpublished results). The average RMSD values for the C^α^ atoms (0.416 Å) and all atoms (0.636 Å) show that, overall, our insulin model superimposed very well with all three insulin structures, with slight differences observed in the solvent-exposed regions, as ex­pected.

### 3.3. Site-specific radiation damage

Site-specific radiation damage affects primarily the C—S bonds in cysteine and methio­nine residues, di­sulfide bonds and the carboxyl­ate groups of negatively charged residues of Asp and Glu (Weik *et al.*, 2000 ▸). To assess the site-specific radiation damage, two independent approaches were followed, *i.e.* *B*_Damage_ ratio analysis (Gerstel *et al.*, 2015 ▸) and di­sulfide bond analysis.

#### 3.3.1. *B*_Damage_ ratio analysis

**Figure 4:**
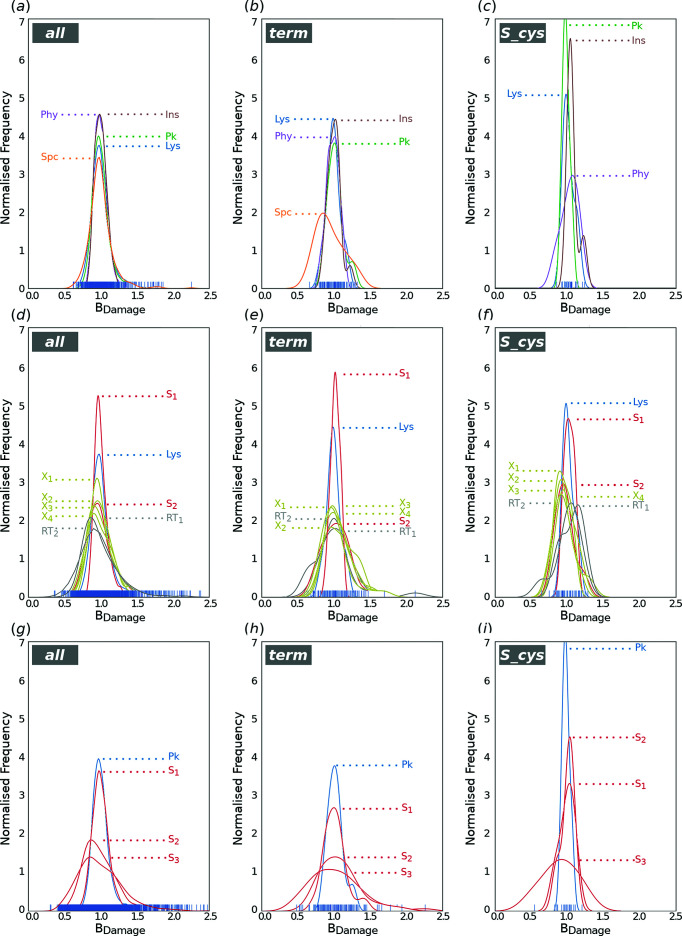
*B*_Damage_ analysis. Distribution of protein structures of those obtained in the present work (top) and of two comparisons of the lysozyme (middle) and proteinase K (bottom) structures with other reference structures considering all protein atoms [*all*; parts (*a*), (*d*) and (*g*)], the terminal GLU Oɛ, ASP Oδ and CYS Sγ atoms [*term*; parts (*b*), (*e*) and (*h*)] or only CYS Sγ atoms [*S_cys*; parts (*c*), (*f*) and (*i*)]. In the top panels [parts (*a*), (*b*) and (*c*)], lysozyme (Lys) is depicted in blue, phycocianin (Phy) in purple, proteinase K (Pk) in green, spectrin C (Spc) in orange and insulin (Ins) in brown. The lysozyme (middle) and proteinase K (bottom) structures of this article are shown in blue and the reference structures are shown in red; other SSX structures (Martin-Garcia, 2021 ▸) collected with a high-viscosity injector at other synchrotron beamlines (middle: S_1_ 4rlm and S_2_ 5uvj; bottom: S_1_ 5uvl, S_2_ 6fjs and S_3_ 6mh6) are shown in red and XFEL structures (middle: X_1_ 5dm9, X_2_ 6h0k, X_3_ 7byo and X_4_ 6h0l) are shown in yellow, while synchrotron structures collected at room tem­per­ature in oscillation mode are shown in grey (middle: RT_1_ 1iee and RT_2_ 6qqe).

*B*_Damage_ is a metric that identifies potential sites of specific damage of atoms with *B*-factor values relative to neighbouring atoms that occupy a similar packing density environment in the crystalline structure (Gerstel *et al.*, 2015 ▸). In this work, we used the procedure described by Gerstel *et al.* (2015 ▸) to calculate the *B*_Damage_ distributions of all protein atoms (*all*), the carboxyl­ate atoms of Glu and Asp residues (*term*), and the S atoms of Cys residues (*S_cys*), to verify if the structures of this study could be prone to radiation damage (Fig. 4 ▸). In order to cross-check the validity of the procedure followed here, a set of protein structures (Nanao *et al.*, 2005 ▸; Sutton *et al.*, 2013 ▸) were used for reference (Fig. S6 in the supporting information). The *B*_Damage_ distribution profiles of *all*, *term*, and *S_cys* atoms for the five proteins collected in this work [Figs. 4 ▸(*a*), 4 ▸(*b*) and 4 ▸(*c*), respectively] are narrower than those from the reference data sets and have higher peaks, which results in smaller variances (Table S3), a reduced data spread and median values closer to 1.0. According to Gerstel and co-workers, these observations are compatible with nonexistent radiation damage, with the sole exception for insulin. This protein presents a high and narrow *B*_Damage_ profile (*v* = 0.006) for the *S_cys* atoms [Fig. 4 ▸(*c*)], in which only two S atoms (corresponding to a single and most labile di­sulfide bond) bear higher *B*_Damage_ values (noticeable as a second subpeak shifted towards higher *B*_Damage_ values) indicative of incipient damage effects, which could be attributed to the fact that insulin data were collected at a lower energy (9.7 keV) instead of 12.6 keV as for the others. At this lower energy, more X-ray dose is deposited into the crystals (Holton, 2009 ▸; Murray *et al.*, 2004 ▸), thus increasing the probability of radiation damage. In this regard, the endogenous presence of the Zn^2+^ ion in the insulin molecule leads to an increased absorbed dose (Garman & Owen, 2006 ▸), specially at its absorption peak, making it highly sensitive to radiation damage. In the case of phycocyanin, the *S_cys* distribution broadening (*v* = 0.014) could be attributed to the relatively high refinement statistics (Table 1 ▸), which may have contributed to cumulative errors in the *B*_Damage_ calculation, as RABDAM is highly sensitive to measured *B*-factors (Gerstel *et al.*, 2015 ▸). As for lysozyme, the *B*_Damage_ distribution of the structure of this work was compared with that of other lysozyme structures collected at room tem­per­ature at synchrotron sources both using SSX and the oscillation method, as well as with lysozyme structures collected at XFELs (Fig. 4 ▸). Our structure presents the second lowest *B*_Damage_ profile variances (Table S3 in the supporting information) for the *all* (*v* = 0.021), *term* (*v* = 0.009) and *S_cys* (*v* = 0.006) atoms, with high peaks closer to *B*_Damage_ values of 1.0, and a small data spread. A similar behaviour, but with the lowest variances, was observed for the comparison of our proteinase K structure with other proteinase K structures determined by the SSX approach (Fig. 4 ▸). In summary, neither the lysozyme nor the proteinase K structure from this work are likely to present significant symptoms of radiation damage, confirming that the SSX structures collected at BL13-XALOC are generally unaffected by X-ray dose.

#### 3.3.2. Di­sulfide bond preferential specific damage

As mentioned above, the specific damage at di­sulfide bonds has been used to qualitatively evaluate the extent of site-specific radiation damage during diffraction experiments through the inspection of difference maps and by integrating different densities in the maps derived from a series of data sets with increments in the incident X-ray dose (de la Mora *et al.*, 2020 ▸; Weik *et al.*, 2000 ▸). However, when the differences in the dose are small and the data are measured using distinct X-ray sources with widely different flux, beam size and divergence, comparisons of densities are difficult to apply. Thus, we probed whether the breakage of a cysteine bond can be followed by comparing the *B*-factor of the S_γ_ atom with the C_β_ atoms. Fig. S7(*d*) (see supporting information) shows the ratio of the *B*-factor of the S_γ_ atom with respect to the C_β_ atom in the cysteines involved in the di­sulfide bonds of the acetyl­cholinesterase in the PDB structures collected at different doses as reported previously (Weik *et al.*, 2000 ▸), as refined by the PDB-REDO database (Joosten *et al.*, 2009 ▸), which reconstructed the S_γ_ atom initially left out of the refinement for the purpose of the difference density analysis. This analysis shows that radiation damage is strongly correlated with the dose at low doses, whereas at high doses, the complete breakage of the bond leads to a plateau [Fig. S7(*d*)]. This plateau is likely related to the restraints used during refinement, which couples the *B*-factors of adjacent atoms such that these do not diverge excessively. In addition, we compared the dose and relative *B*-factors of the acetylcholine (ACh) structures with other relevant PDB structures analyzed herein and find again a good correlation of the relative *B*-factor with dose [Fig. S7(*d*)]. The exception was the structure of PDB entry 6qqe (Gotthard *et al.*, 2019 ▸), which was collected with low dose at room tem­per­ature using a standard single-sweep data collection protocol. The relatively high *B*-factor can be explained by the fact that it took 10 s to collect the associated data set on a single crystal, during which the breakage of the di­sulfide bond was allowed to develop. In contrast, the data described in our study were collected from individual microcrystals in which each crystal was illuminated with X-rays for just 100 ms, thus avoiding the propagation of radiation damage in the crystals.

Figs. S7(*a*)–7(*c*) (see supporting information) show the ratio of tem­per­ature *B*-factor values for the S_γ_/C_β_ atoms of cysteines involved in the di­sulfide bonds calculated as described in the *Materials and methods* section (§2) for the lysozyme, proteinase K and insulin structures presented herein, and for several structures deposited in the PDB, *i.e.* 6qqe (Gotthard *et al.*, 2019 ▸), 1qio (Weik *et al.*, 2000 ▸), 1iee (Sauter *et al.*, 2001 ▸) and 6ftr (Wiedorn *et al.*, 2018 ▸) for lysozyme, 2pwa (unpublished results), 5kxu (Masuda *et al.*, 2017 ▸) and 1ic6 (Betzel *et al.*, 2001 ▸) for proteinase K, and 4f1g (Fávero-Retto *et al.*, 2013 ▸) and 5e7w (Lisgarten *et al.*, 2017 ▸) for insulin. These PDBs were selected based on the relevance of the experimental conditions at which the crystal structures were collected, such as the tem­per­ature of data collection, the sample delivery method used and the X-ray radiation source.

The relative *B*-factors of the structures described herein show significantly lower values compared to the analyzed published structures, suggesting that radiation damage is reduced. For example, data collected at extremely brilliant XFELs using a jet stream (PDB entry 5kxu; Masuda *et al.*, 2017 ▸) showed a significant increase in the *B*-factors of the S atoms, indicating that site-specific radiation damage occurs. Thus, we conclude that SSX with viscous jets at synchrotrons, and in particular at BL13-XALOC, can provide data with minimal radiation damage.

## 4. Conclusions and future directions

The advantages that microcrystals offer over their larger counterparts has triggered the desire to perform SSX at synchrotrons, resulting in a high demand from the user community. This is leading to the accelerated development of beamlines dedicated to this approach worldwide. To advance the MX capabilities at ALBA, the MX beamline BL13-XALOC has established and included the SSX approach with viscous jets as a new method in its data collection pipeline, which can be tailored to the daily crystallographic and structural biology problems our users bring to the beamline. We have established the feasibility of per­forming SSX experiments at BL13-XALOC by determining high-resolution protein structures, demonstrating that the flux and the size of the focus resulted in adequate data quality. SSX with viscous jets offers a variety of advantages over standard crystallography such as: (1) solving structures in those cases where obtaining large crystals is an issue; (2) collecting room-tem­per­ature measurements, which allow us to detect and visualize functionally relevant molecular changes; (3) gaining access to ligand-binding pockets, which facilitates binding studies and fragment-based drug discovery; (4) providing electronic and spatial features to aid in the modelling of new drug-like compounds; (5) performing derivatization experiments without the need of fishing crystals; and (6) mitigating radiation damage. Based on this, it should now be feasible to conduct time-resolved pump–probe measurements to study light-induced reactions on millisecond timescales from microcrystals using the existing infrastructure at BL13-XALOC.

The upcoming ALBA II upgrade will bring this light source from a third-generation to a fourth-generation synchrotron, producing a much brighter and more coherent photon X-ray beam. The implementation of the SSX strategy will improve with the future upgrades at ALBA II, which will create a smaller brighter micro-focused beam with an intensity that is expected to increase by several orders of magnitude. These improvements, along with new developments in beamline optics and the acquisition of a faster frame-readout detector, will allow BL13-XALOC to determine macromolecular crystal structures from smaller crystals. Also, we anticipate that the increase in signal-to-noise ratio will allow a decrease of the repetition rate into the low microsecond range after the upcoming upgrade.

The structural biology program at ALBA comprises the development of a microfocus MX beamline called BL-06-XAIRA, currently in the construction phase (https://www.cells.es/en/beamlines/bl06-xaira. XAIRA will be dedicated to oscillation-based experiments and fixed-target serial crystallography, and will complement the scientific case of the BL13-XALOC beamline. The combination of a beam size down to 1 µm × 1 µm, together with the fixed target and jet approaches, will open the possibility to perform time-resolved studies with microsecond resolution ensuring that BL06-XAIRA will help to expand the toolbox of the Spanish SSX user community. The construction of BL06-XAIRA has started recently and the first users will be hosted during 2023.

Radiation damage has been a limiting factor in obtaining high-quality protein structures in standard X-ray crystallography at synchrotron sources. However, nowadays, radiation damage can be substantially mitigated by the adaptation of the SSX approach, in which the crystal structures of macromolecules are solved from data sets collected from thousands of crystals. After SSX came into play, over 40 experiments have been conducted (Martin-Garcia, 2021 ▸) and, to the best of our knowledge, site-specific radiation damage has only been reported for the di­sulfide bridges of lysozyme from a data set in which a much higher dose than the theoretical safe dose limit of 0.3 MGy (Nave & Garman, 2005 ▸) was reached (Coquelle *et al.*, 2015 ▸). In the study presented here, the radiation dose estimated by RADDOSE 3D never went above the previously reported dose limit, which indicated that site-specific radiation damage was not significant for all protein structures but insulin, and, therefore, the integrity of the structures was not affected. This was also supported by the *B*_Damage_ values presented in Fig. 4 ▸. It is largely known that proteins containing redox cofactors or heavy elements in their structure are particularly more sensitive to radiation damage due to the increased probability of interaction with the X-rays. Thus, it has been reported that heavy atoms and the atoms in their proximity are areas of increased localized structural and electronic damage compared to the rest of the atoms in a protein structure (Hau-Riege *et al.*, 2004 ▸; Jurek & Faigel, 2009 ▸). In conclusion, offering SSX approaches at MX synchrotron beamlines is an important addition, offering users the option of collecting very low dose data sets even for microcrystals.

## Supplementary Material

Supplemental Information figures S1 to S7, Tables S1 to S3. DOI: 10.1107/S1600577522002508/rv5160sup1.pdf

Structure factors: contains datablock(s) rxxxxsf. DOI: 10.1107/S1600577522002508/rv5160sup2.hkl

Structure factors: contains datablock(s) rxxxxsf. DOI: 10.1107/S1600577522002508/rv5160sup3.hkl

Structure factors: contains datablock(s) rxxxxsf. DOI: 10.1107/S1600577522002508/rv5160sup4.hkl

Structure factors: contains datablock(s) rxxxxsf. DOI: 10.1107/S1600577522002508/rv5160sup5.hkl

Structure factors: contains datablock(s) rxxxxsf. DOI: 10.1107/S1600577522002508/rv5160sup6.hkl

## Figures and Tables

**Table 1:** Data collection statistics (values in parentheses are for the high-resolution shell)

	Lysozyme	Proteinase K	Phycocyanin	α-Spectrin-SH3	Insulin
Beamline	BL13 XALOC	BL13 XALOC	BL13 XALOC	BL13 XALOC	BL13 XALOC
Wavelength (Å)	0.98	0.98	0.98	0.98	1.28
Temperature (K)	295	295	295	295	295
Crystal size (µm)	10 × 10 × 5	15 × 10 × 5	20 × 15 × 5	30 × 5 × 5	20 × 30 × 30
Flow rate (nl min^−1^)	29.4	29.4	29.4	71.4	71.4
Exposure time (ms)	80	80	80	80	80
Data collection time (h)	2.6	11	17	3	7
Sample consumption (µl)	4.5	19.4	30	14	30
Max. dose per crystal (KGy)	115	115	125	98.4	150
Crystal-detector distance (mm)	506	506	506	506	506
No. of images collected	114080	577904	753533	139971	433986
No. of indexed patterns	23733	216645	152142	13039	51144
Space group	*P*4_3_2_1_2	*P*4_3_2_1_2	*H*3_2_	*P*2_1_2_1_2_1_	*H*3
*a*, *b*, *c* (Å)	79.00, 79.00, 38.2	68.40, 68.40, 108.4	188.5, 188.5, 61.0	34.2, 42.6, 50.8	81.6, 81.6, 33.6
α, β, γ (°)	90, 90, 90	90, 90, 90	90, 90, 120	90, 90, 90	90, 90, 420
Resolution range (Å)	38.2–2.1 (2.17–2.1)	48.4–1.9 (1.97–1.9)	48.8–2.1 (2.17–2.1)	42.6–2.1 (2.17–2.1)	30.3–1.7 (1.76–1.7)
Total No. of reflections	5089514 (115198)	78935391 (640218)	72742152 (1687827)	1145735 (25647)	11811721 (817546)
No. of unique reflections	7498 (716)	21010 (2033)	24059 (2370)	4682 (454)	9140 (928)
Completeness (%)	100 (100)	100 (100)	100 (100)	100 (100)	100 (100)
Redundancy	679 (161)	3757 (315)	3023 (712)	245 (57)	1292 (881)
〈*I*/σ(*I*)〉	9.8 (0.5)	17.4 (0.6)	12.6 (0.6)	6.5 (0.3)	7.9 (1.5)
CC* (%)	99.90 (58.55)	99.96 (60.67)	99.92 (29.60)	99.75 (58.17)	99.47 (73.28)
*R*_split_	7.6 (157.9)	4.6 (145.1)	7.7 (231.1)	10.1 (229.7)	12.3 (74.8)
Wilson *B*-factor (Å^2^)	37.4	23.7	42.3	50.3	29.1

**Table 2:** Data refinement statistics (values in parentheses are for the high-resolution shell)

	Lysozyme	Proteinase K	Phycocyanin	α-Spectrin-SH3	Insulin
Resolution range (Å)	35.35–2.1 (2.16–2.10)	44.21–1.9 (1.95–1.9)	43.4–2.1 (2.16–2.1)	32.6–2.1 (2.16–2.1)	24.35–1.71
Completeness (%)	100 (100)	100 (100)	99.2 (90.0)	99.1 (88.1)	99.7 (96.0)
No. of reflections, working set	6723 (479)	19879 (1431)	22830 (1530)	4110 (264)	8163 (584)
No. of reflections, test set	741 (59)	1071 (79)	1185 (78)	503 (33)	908 (56)
R_work_ (%)	17.9	16.5	30.5	19.4	24.0
R_free_ (%)	23.2	19.6	34.7	23.9	25.8
No. of atoms					
Protein	1001	2068	2488	462	808
Ions	1	2	2	0	3
Ligands	0	4	138	0	0
Water	17	89	43	5	15
Total	1019	2163	2671	467	826
R.m.s. deviations					
Bonds (Å)	0.010	0.011	0.005	0.012	0.006
Angles (°)	1.582	1.596	1.162	1.826	1.271
Average *B*-factors (Å^2^)					
Protein	46.9	30.4	53.9	62.1	33.0
Ions	64.5	33.0	92.8	0	35.1
Ligands	0	55.3	57.6	0	0
Water	40.0	35.1	47.4	59.7	34.2
Ramachandran plot					
Favoured (%)	96.1	96.0	97.6	98.2	94.7
Allowed (%)	3.9	3.6	2.1	1.8	4.3
PDB code	7s4w	7s4z	7s4z	7s4r	7s4y

## References

[bb1] Artusio, F., Castellví, A., Sacristán, A., Pisano, R. & Gavira, J. A. (2020). *Cryst. Growth Des.* **20**, 5564–5571.

[bb2] Betzel, C., Gourinath, S., Kumar, P., Kaur, P., Perbandt, M., Eschenburg, S. & Singh, T. P. (2001). *Biochemistry*, **40**, 3080–3088. 10.1021/bi002538n 11258922

[bb3] Botha, S., Baitan, D., Jungnickel, K. E. J., Oberthür, D., Schmidt, C., Stern, S., Wiedorn, M. O., Perbandt, M., Chapman, H. N. & Betzel, C. (2018). *IUCrJ*, **5**, 524–530. 10.1107/S2052252518009223 PMC6126645 30224955

[bb4] Botha, S., Nass, K., Barends, T. R. M., Kabsch, W., Latz, B., Dworkowski, F., Foucar, L., Panepucci, E., Wang, M., Shoeman, R. L., Schlichting, I. & Doak, R. B. (2015). *Acta Cryst.* D**71**, 387–397. 10.1107/S1399004714026327 25664750

[bb5] Cámara-Artigas, A., Gavira, J. A., Casares, S., Garcia-Ruiz, J. M., Conejero-Lara, F., Allen, J. P. & Martinez, J. C. (2011). *Acta Cryst.* D**67**, 189–196. 10.1107/S0907444911001715 21358049

[bb6] Colldelram, C., Ruget, C. & Nikitina, L. (2010). *DLS Proc.* **1**, e44.

[bb7] Coquelle, N., Brewster, A. S., Kapp, U., Shilova, A., Weinhausen, B., Burghammer, M. & Colletier, J.-P. (2015). *Acta Cryst.* D**71**, 1184–1196. 10.1107/S1399004715004514 PMC4427202 25945583

[bb8] Duisenberg, A. J. M. (1992). *J. Appl. Cryst.* **25**, 92–96.

[bb9] Emsley, P., Lohkamp, B., Scott, W. G. & Cowtan, K. (2010). *Acta Cryst.* D**66**, 486–501. 10.1107/S0907444910007493 PMC2852313 20383002

[bb10] Evans, P. R. (2011). *Acta Cryst.* D**67**, 282–292. 10.1107/S090744491003982X PMC3069743 21460446

[bb11] Evans, P. R. & Murshudov, G. N. (2013). *Acta Cryst.* D**69**, 1204–1214. 10.1107/S0907444913000061 PMC3689523 23793146

[bb12] Fávero-Retto, M. P., Palmieri, L. C., Souza, T. A. C. B., Almeida, F. C. L. & Lima, L. M. T. R. (2013). *Eur. J. Pharm. Biopharm.* **85**, 1112–1121. 10.1016/j.ejpb.2013.05.005 23692694

[bb13] Fromme, R., Ishchenko, A., Metz, M., Chowdhury, S. R., Basu, S., Boutet, S., Fromme, P., White, T. A., Barty, A., Spence, J. C. H., Weierstall, U., Liu, W. & Cherezov, V. (2015). *IUCrJ*, **2**, 545–551. 10.1107/S2052252515013160 PMC4547822 26306196

[bb14] Garman, E. F. & Owen, R. L. (2006). *Acta Cryst.* D**62**, 32–47. 10.1107/S0907444905034207 16369092

[bb15] Gerstel, M., Deane, C. M. & Garman, E. F. (2015). *J. Synchrotron Rad.* **22**, 201–212. 10.1107/S1600577515002131 PMC4344357 25723922

[bb16] Gevorkov, Y., Yefanov, O., Barty, A., White, T. A., Mariani, V., Brehm, W., Tolstikova, A., Grigat, R.-R. & Chapman, H. N. (2019). *Acta Cryst.* A**75**, 694–704. 10.1107/S2053273319010593 PMC6718201 31475914

[bb17] Goddard, T. D., Huang, C. C., Meng, E. C., Pettersen, E. F., Couch, G. S., Morris, J. H. & Ferrin, T. E. (2018). *Protein Sci.* **27**, 14–25. 10.1002/pro.3235 PMC5734306 28710774

[bb18] Gotthard, G., Aumonier, S., De Sanctis, D., Leonard, G., von Stetten, D. & Royant, A. (2019). *IUCrJ*, **6**, 665–680. 10.1107/S205225251900616X PMC6608634 31316810

[bb19] Hau-Riege, S. P., London, R. A. & Szoke, A. (2004). *Phys. Rev. E*, **69**, 051906. 10.1103/PhysRevE.69.051906 15244846

[bb20] Holton, J. M. (2009). *J. Synchrotron Rad.* **16**, 133–142.

[bb21] Horrell, S., Axford, D., Devenish, N. E., Ebrahim, A., Hough, M. A., Sherrell, D. A., Storm, S. L. S., Tews, I., Worrall, J. A. R. & Owen, R. L. (2021). *J. Vis. Exp.* **168**, e62200. 10.3791/62200 33720136

[bb22] Joosten, R. P., Salzemann, J., Bloch, V., Stockinger, H., Berglund, A.-C., Blanchet, C., Bongcam-Rudloff, E., Combet, C., Da Costa, A. L., Deleage, G., Diarena, M., Fabbretti, R., Fettahi, G., Flegel, V., Gisel, A., Kasam, V., Kervinen, T., Korpelainen, E., Mattila, K., Pagni, M., Reichstadt, M., Breton, V., Tickle, I. J. & Vriend, G. (2009). *J. Appl. Cryst.* **42**, 376–384. 10.1107/S0021889809008784 PMC3246819 22477769

[bb24] Juanhuix, J., González, N., Garriga, D., Campmany, J., Marcos, J., Nikitina, L., Colldelram, C. & Nicolas, J. (2019). *AIP Conf. Proc.* **2054**, 060032.

[bb23] Juanhuix, J., Gil-Ortiz, F., Cuní, G., Colldelram, C., Nicolás, J., Lidón, J., Boter, E., Ruget, C., Ferrer, S. & Benach, J. (2014). *J. Synchrotron Rad.* **21**, 679–689. 10.1107/S160057751400825X PMC4073956 24971961

[bb25] Jurek, Z. & Faigel, G. (2009). *Europhys. Lett.* **86**, 68003.

[bb26] Kabsch, W. (1976). *Acta Cryst.* A**32**, 922–923.

[bb27] Kabsch, W. (2010). *Acta Cryst.* D**66**, 125–132. 10.1107/S0907444909047337 PMC2815665 20124692

[bb28] Kirkpatrick, P. & Baez, A. V. (1948). *J. Opt. Soc. Am.* **38**, 766–774. 10.1364/josa.38.000766 18883922

[bb943] Kovalev, K., Astashkin, R., Gushchin, I., Orekhov, P., Volkov, D., Zinovev, E., Marin, E., Rulev, M., Alekseev, A., Royant, A., Carpentier, P., Vaganova, S., Zabelskii, D., Baeken, C., Sergeev, I., Balandin, T., Bourenkov, G., Carpena, X., Boer, R., Maliar, N., Borshchevskiy, V., Büldt, G., Bamberg, E. & Gordeliy, V. (2020). *Nat Commun*, **11**, 2137. 10.1038/s41467-020-16032-y PMC7195465 32358514

[bb29] Kurochkina, N. & Guha, U. (2013). *Biophys. Rev.* **5**, 29–39. 10.1007/s12551-012-0081-z PMC5418429 28510178

[bb30] Lebedev, A. A., Vagin, A. A. & Murshudov, G. N. (2008). *Acta Cryst.* D**64**, 33–39. 10.1107/S0907444907049839 PMC2394799 18094465

[bb31] Lisgarten, D. R., Palmer, R. A., Lobley, C. M. C., Naylor, C. E., Chowdhry, B. Z., Al-Kurdi, Z. I., Badwan, A. A., Howlin, B. J., Gibbons, N. C. J., Saldanha, J. W., Lisgarten, J. N. & Basak, A. K. (2017). *Chem. Cent. J.* **11**, 73. 10.1186/s13065-017-0296-y PMC5539060 29086855

[bb32] Martin-Garcia, J. M. (2021). *Crystals*, **11**, 521.

[bb33] Martin-Garcia, J. M., Conrad, C. E., Nelson, G., Stander, N., Zatsepin, N. A., Zook, J., Zhu, L., Geiger, J., Chun, E., Kissick, D., Hilgart, M. C., Ogata, C., Ishchenko, A., Nagaratnam, N., Roy-Chowdhury, S., Coe, J., Subramanian, G., Schaffer, A., James, D., Ketwala, G., Venugopalan, N., Xu, S., Corcoran, S., Ferguson, D., Weierstall, U., Spence, J. C. H., Cherezov, V., Fromme, P., Fischetti, R. F. & Liu, W. (2017). *IUCrJ*, **4**, 439–454. 10.1107/S205225251700570X PMC5571807 28875031

[bb34] Martin-Garcia, J. M., Zhu, L., Mendez, D., Lee, M.-Y., Chun, E., Li, C., Hu, H., Subramanian, G., Kissick, D., Ogata, C., Henning, R., Ishchenko, A., Dobson, Z., Zhang, S., Weierstall, U., Spence, J. C. H., Fromme, P., Zatsepin, N. A., Fischetti, R. F., Cherezov, V. & Liu, W. (2019). *IUCrJ*, **6**, 412–425. 10.1107/S205225251900263X PMC6503920 31098022

[bb35] Masuda, T., Suzuki, M., Inoue, S., Song, C., Nakane, T., Nango, E., Tanaka, R., Tono, K., Joti, Y., Kameshima, T., Hatsui, T., Yabashi, M., Mikami, B., Nureki, O., Numata, K., Iwata, S. & Sugahara, M. (2017). *Sci. Rep.* **7**, 45604. 10.1038/srep45604 PMC5374539 28361898

[bb36] Meents, A., Wiedorn, M. O., Srajer, V., Henning, R., Sarrou, I., Bergtholdt, J., Barthelmess, M., Reinke, P. Y. A., Dierksmeyer, D., Tolstikova, A., Schaible, S., Messerschmidt, M., Ogata, C. M., Kissick, D. J., Taft, M. H., Manstein, D. J., Lieske, J., Oberthuer, D., Fischetti, R. F. & Chapman, H. N. (2017). *Nat. Commun.* **8**, 1281. 10.1038/s41467-017-01417-3 PMC5668288 29097720

[bb37] Mehrabi, P., Müller-Werkmeister, H. M., Leimkohl, J.-P., Schikora, H., Ninkovic, J., Krivokuca, S., Andriček, L., Epp, S. W., Sherrell, D., Owen, R. L., Pearson, A. R., Tellkamp, F., Schulz, E. C. & Miller, R. J. D. (2020). *J. Synchrotron Rad.* **27**, 360–370. 10.1107/S1600577520000685 PMC7064102 32153274

[bb38] Mehrabi, P., Schulz, E. C., Dsouza, R., Müller-Werkmeister, H. M., Tellkamp, F., Miller, R. J. D. & Pai, E. F. (2019). *Science*, **365**, 1167–1170. 10.1126/science.aaw9904 31515393

[bb39] Mora, E. de la, Coquelle, N., Bury, C. S., Rosenthal, M., Holton, J. M., Carmichael, I., Garman, E. F., Burghammer, M., Colletier, J.-P. & Weik, M. (2020). *Proc. Natl Acad. Sci.* **117**, 4142–4151. 10.1073/pnas.1821522117 PMC7049125 32047034

[bb40] Murray, J. W., Garman, E. F. & Ravelli, R. B. G. (2004). *J. Appl. Cryst.* **37**, 513–522.

[bb41] Murray, T. D., Lyubimov, A. Y., Ogata, C. M., Vo, H., Uervirojnangkoorn, M., Brunger, A. T. & Berger, J. M. (2015). *Acta Cryst.* D**71**, 1987–1997. 10.1107/S1399004715015011 PMC4601365 26457423

[bb42] Murshudov, G. N., Skubák, P., Lebedev, A. A., Pannu, N. S., Steiner, R. A., Nicholls, R. A., Winn, M. D., Long, F. & Vagin, A. A. (2011). *Acta Cryst.* D**67**, 355–367. 10.1107/S0907444911001314 PMC3069751 21460454

[bb43] Nanao, M. H., Sheldrick, G. M. & Ravelli, R. B. G. (2005). *Acta Cryst.* D**61**, 1227–1237. 10.1107/S0907444905019360 16131756

[bb44] Nave, C. & Garman, E. F. (2005). *J. Synchrotron Rad.* **12**, 257–260. 10.1107/S0909049505007132 15840908

[bb45] Nogly, P., James, D., Wang, D., White, T. A., Zatsepin, N., Shilova, A., Nelson, G., Liu, H., Johansson, L., Heymann, M., Jaeger, K., Metz, M., Wickstrand, C., Wu, W., Båth, P., Berntsen, P., Oberthuer, D., Panneels, V., Cherezov, V., Chapman, H., Schertler, G., Neutze, R., Spence, J., Moraes, I., Burghammer, M., Standfuss, J. & Weierstall, U. (2015). *IUCrJ*, **2**, 168–176. 10.1107/S2052252514026487 PMC4392771 25866654

[bb46] Pettersen, E. F., Goddard, T. D., Huang, C. C., Couch, G. S., Greenblatt, D. M., Meng, E. C. & Ferrin, T. E. (2004). *J. Comput. Chem.* **25**, 1605–1612. 10.1002/jcc.20084 15264254

[bb47] Powell, H. R., Johnson, O. & Leslie, A. G. W. (2013). *Acta Cryst.* D**69**, 1195–1203. 10.1107/S0907444912048524 PMC3689522 23793145

[bb48] Roedig, P., Vartiainen, I., Duman, R., Panneerselvam, S., Stübe, N., Lorbeer, O., Warmer, M., Sutton, G., Stuart, D. I., Weckert, E., David, C., Wagner, A. & Meents, A. (2015). *Sci. Rep.* **5**, 10451. 10.1038/srep10451 PMC4448500 26022615

[bb49] Sadqi, M., Casares, S., Abril, M. A., López-Mayorga, O., Conejero-Lara, F. & Freire, E. (1999). *Biochemistry*, **38**, 8899–8906. 10.1021/bi990413g 10413463

[bb50] Sauter, C., Otálora, F., Gavira, J.-A., Vidal, O., Giegé, R. & García-Ruiz, J. M. (2001). *Acta Cryst.* D**57**, 1119–1126. 10.1107/s0907444901008873 11468395

[bb51] Schulz, E. C., Mehrabi, P., Müller-Werkmeister, H. M., Tellkamp, F., Jha, A., Stuart, W., Persch, E., De Gasparo, R., Diederich, F., Pai, E. F. & Miller, R. J. D. (2018). *Nat. Methods*, **15**, 901–904. 10.1038/s41592-018-0180-2 30377366

[bb52] Shelley, K. L., Dixon, T. P. E., Brooks-Bartlett, J. C. & Garman, E. F. (2018). *J. Appl. Cryst.* **51**, 552–559. 10.1107/S1600576718002509 PMC5884390 29657569

[bb53] Smith, G. D. & Blessing, R. H. (2003). *Acta Cryst.* D**59**, 1384–1394. 10.1107/s090744490301165x 12876340

[bb54] Sutton, K. A., Black, P. J., Mercer, K. R., Garman, E. F., Owen, R. L., Snell, E. H. & Bernhard, W. A. (2013). *Acta Cryst.* D**69**, 2381–2394. 10.1107/S0907444913022117 PMC3852651 24311579

[bb55] Vagin, A. & Teplyakov, A. (2010). *Acta Cryst.* D**66**, 22–25. 10.1107/S0907444909042589 20057045

[bb56] Weierstall, U., James, D., Wang, C., White, T. A., Wang, D., Liu, W., Spence, J. C. H., Bruce Doak, R., Nelson, G., Fromme, P., Fromme, R., Grotjohann, I., Kupitz, C., Zatsepin, N. A., Liu, H., Basu, S., Wacker, D., Won Han, G., Katritch, V., Boutet, S., Messerschmidt, M., Williams, G. J., Koglin, J. E., Marvin Seibert, M., Klinker, M., Gati, C., Shoeman, R. L., Barty, A., Chapman, H. N., Kirian, R. A., Beyerlein, K. R., Stevens, R. C., Li, D., Shah, S. T. A., Howe, N., Caffrey, M. & Cherezov, V. (2014). *Nat. Commun.* **5**, 3309. 10.1038/ncomms4309 PMC4061911 24525480

[bb57] Weik, M., Ravelli, R. B. G., Kryger, G., McSweeney, S., Raves, M. L., Harel, M., Gros, P., Silman, I., Kroon, J. & Sussman, J. L. (2000). *Proc. Natl Acad. Sci. USA*, **97**, 623–628. 10.1073/pnas.97.2.623 PMC15380 10639129

[bb58] White, T. A. (2019). *Acta Cryst.* D**75**, 219–233. 10.1107/S205979831801238X PMC6400257 30821710

[bb59] White, T. A., Kirian, R. A., Martin, A. V., Aquila, A., Nass, K., Barty, A. & Chapman, H. N. (2012). *J. Appl. Cryst.* **45**, 335–341.

[bb60] Wiedorn, M. O., Oberthür, D., Bean, R., Schubert, R., Werner, N., Abbey, B., Aepfelbacher, M., Adriano, L., Allahgholi, A., Al-Qudami, N., Andreasson, J., Aplin, S., Awel, S., Ayyer, K., Bajt, S., Barák, I., Bari, S., Bielecki, J., Botha, S., Boukhelef, D., Brehm, W., Brockhauser, S., Cheviakov, I., Coleman, M. A., Cruz-Mazo, F., Danilevski, C., Darmanin, C., Doak, R. B., Domaracky, M., Dörner, K., Du, Y., Fangohr, H., Fleckenstein, H., Frank, M., Fromme, P., Gañán-Calvo, A. M., Gevorkov, Y., Giewekemeyer, K., Ginn, H. M., Graafsma, H., Graceffa, R., Greiffenberg, D., Gumprecht, L., Göttlicher, P., Hajdu, J., Hauf, S., Heymann, M., Holmes, S., Horke, D. A., Hunter, M. S., Imlau, S., Kaukher, A., Kim, Y., Klyuev, A., Knoška, J., Kobe, B., Kuhn, M., Kupitz, C., Küpper, J., Lahey-Rudolph, J. M., Laurus, T., Le Cong, K., Letrun, R., Xavier, P. L., Maia, L., Maia, F. R. N. C., Mariani, V., Messerschmidt, M., Metz, M., Mezza, D., Michelat, T., Mills, G., Monteiro, D. C. F., Morgan, A., Mühlig, K., Munke, A., Münnich, A., Nette, J., Nugent, K. A., Nuguid, T., Orville, A. M., Pandey, S., Pena, G., Villanueva-Perez, P., Poehlsen, J., Previtali, G., Redecke, L., Riekehr, W. M., Rohde, H., Round, A., Safenreiter, T., Sarrou, I., Sato, T., Schmidt, M., Schmitt, B., Schönherr, R., Schulz, J., Sellberg, J. A., Seibert, M. M., Seuring, C., Shelby, M. L., Shoeman, R. L., Sikorski, M., Silenzi, A., Stan, C. A., Shi, X., Stern, S., Sztuk-Dambietz, J., Szuba, J., Tolstikova, A., Trebbin, M., Trunk, U., Vagovic, P., Ve, T., Weinhausen, B., White, T. A., Wrona, K., Xu, C., Yefanov, O., Zatsepin, N., Zhang, J., Perbandt, M., Mancuso, A. P., Betzel, C., Chapman, H. & Barty, A. (2018). *Nat. Commun.* **9**, 4025.

[bb987] Winn, M. D., Ballard, C. C., Cowtan, K. D., Dodson, E. J., Emsley, P., Evans, P. R., Keegan, R. M., Krissinel, E. B., Leslie, A. G. W., McCoy, A., McNicholas, S. J., Murshudov, G. N., Pannu, N. S., Potterton, E. A., Powell, H. R., Read, R. J., Vagin, A. & Wilson, K. S. (2011). *Acta Cryst.* D**67**, 235–242. 10.1107/S0907444910045749 PMC3069738 21460441

[bb61] Zeldin, O. B., Brockhauser, S., Bremridge, J., Holton, J. M. & Garman, E. F. (2013). *Proc. Natl Acad. Sci. USA*, **110**, 20551–20556. 10.1073/pnas.1315879110 PMC3870734 24297937

