# Serial macromolecular crystallography at ALBA Synchrotron Light Source. Erratum

**DOI:** 10.1107/S1600577522005185

**Published:** 2022-05-16

**Authors:** Jose M. Martin-Garcia, Sabine Botha, Hao Hu, Rebecca Jernigan, Albert Castellví, Stella Lisova, Fernando Gil, Barbara Calisto, Isidro Crespo, Shatabdi Roy-Chowdhury, Alice Grieco, Gihan Ketawala, Uwe Weierstall, John Spence, Petra Fromme, Nadia Zatsepin, Dirk Roeland Boer, Xavi Carpena

**Affiliations:** a Center for Applied Structural Discovery, Biodesign Institute, Arizona State University, Tempe, AZ, USA; b Department of Crystallography and Structural Biology, Institute of Physical Chemistry Rocasolano, Spanish National Research Council (CSIC), Madrid, Spain; c Department of Physics, Arizona State University, Tempe, AZ, USA; d Molecular Biology Institute of Barcelona, CSIC, Barcelona, Spain; e ALBA Synchrotron, Cerdanyola del Vallès, Barcelona, Spain; f ARC Centre of Excellence in Advance Molecular Physics, La Trobe Institute for Molecular ScienceImaging, Department of Chemistry and Physics, La Trobe University, Melbourne, Australia

**Keywords:** serial synchrotron crystallography, viscous jet, LCP, microcrystal, ALBA, XALOC

## Abstract

A revised version of Table 2 of Martin-Garcia *et al.* [ *J. Synchrotron Rad.* (2022). **29**, 896–907] is provided.

## Abstract 2

A PDB code given in Table 2 of Martin-Garcia *et al.* [ *J. Synchrotron Rad.* (2022). **29**, 896–907] is corrected.

The PDB code for phycocyanin given in Table 2 ▸ in the paper by Martin-Garcia *et al.* (2022 ▸) was incorrectly given as 7s4z. The correct code is 7s50. The full correct table is shown below.

## Figures and Tables

**Table 2:** Data refinement statistics (values in parentheses are for the high-resolution shell)

	Lysozyme	Proteinase K	Phycocyanin	α-Spectrin-SH3	Insulin
Resolution range (Å)	35.35–2.1 (2.16–2.10)	44.21–1.9 (1.95–1.9)	43.4–2.1 (2.16–2.1)	32.6–2.1 (2.16–2.1)	24.35–1.71
Completeness (%)	100 (100)	100 (100)	99.2 (90.0)	99.1 (88.1)	99.7 (96.0)
No. of reflections, working set	6723 (479)	19879 (1431)	22830 (1530)	4110 (264)	8163 (584)
No. of reflections, test set	741 (59)	1071 (79)	1185 (78)	503 (33)	908 (56)
R_work_ (%)	17.9	16.5	30.5	19.4	24.0
R_free_ (%)	23.2	19.6	34.7	23.9	25.8
No. of atoms					
Protein	1001	2068	2488	462	808
Ions	1	2	2	0	3
Ligands	0	4	138	0	0
Water	17	89	43	5	15
Total	1019	2163	2671	467	826
R.m.s. deviations					
Bonds (Å)	0.010	0.011	0.005	0.012	0.006
Angles (°)	1.582	1.596	1.162	1.826	1.271
Average *B*-factors (Å^2^)					
Protein	46.9	30.4	53.9	62.1	33.0
Ions	64.5	33.0	92.8	0	35.1
Ligands	0	55.3	57.6	0	0
Water	40.0	35.1	47.4	59.7	34.2
Ramachandran plot					
Favoured (%)	96.1	96.0	97.6	98.2	94.7
Allowed (%)	3.9	3.6	2.1	1.8	4.3
PDB code	7s4w	7s4z	7s50	7s4r	7s4y
