# Supplementary material for: Serial macromolecular crystallography at ALBA Synchrotron Light Source
Source: J Synchrotron Radiat. 2022 Apr 4;29(Pt 3):896–907. doi: 10.1107/S1600577522002508 (PMC9070724; doi:10.1107/S1600577522002508)
Supplement: Supplementary file 1 [file s-29-00896-sup1.pdf]

JOURNAL OF SYNCHROTRON RADIATION

Volume 29 (2022)

Supporting information for article:

Serial Macromolecular Crystallography at ALBA Synchrotron Light Source

Jose M. Martin-Garcia, Sabine Botha, Hao Hu, Rebecca Jernigan, Albert Castellví, Stella Lisova, Fernando Gil, Barbara Calisto, Isidro Crespo, Shatabdi Roy-Chowdhury, Alice Grieco, Gihan Ketawala, Uwe Weierstall, John Spence, Petra Fromme, Nadia Zatsepin, Roeland Boer and Xavi Carpena

Supplemental Figures

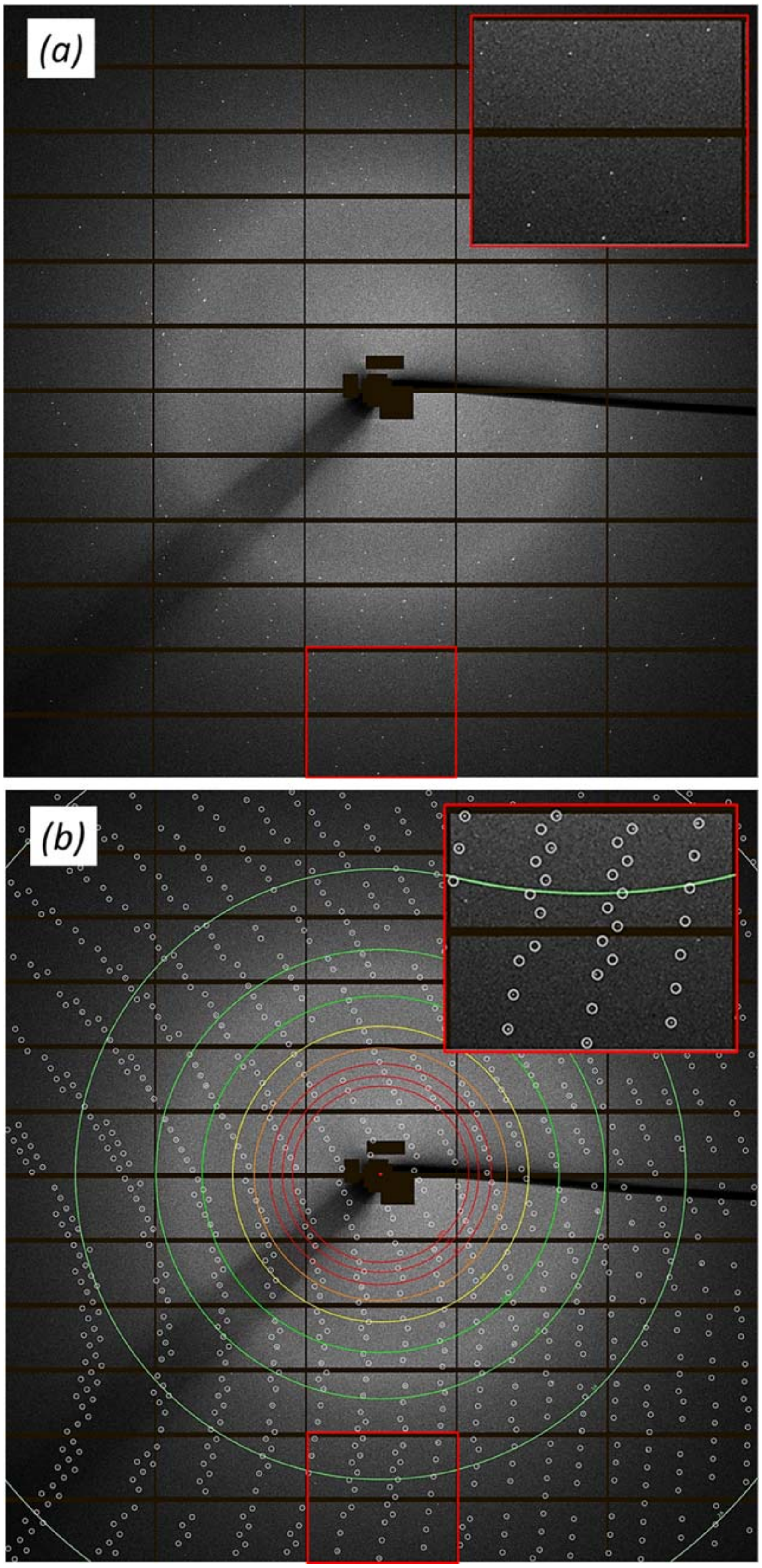

Figure S1. Diffraction pattern of a single micro-crystal of lysozyme suspended in LCP *(a)* Diffraction pattern showing Bragg peaks up to nearly 2 Å resolution. *(b)* Same diffraction pattern in *(a)* after indexing with resolution rings ON (showing the predicted spot locations).

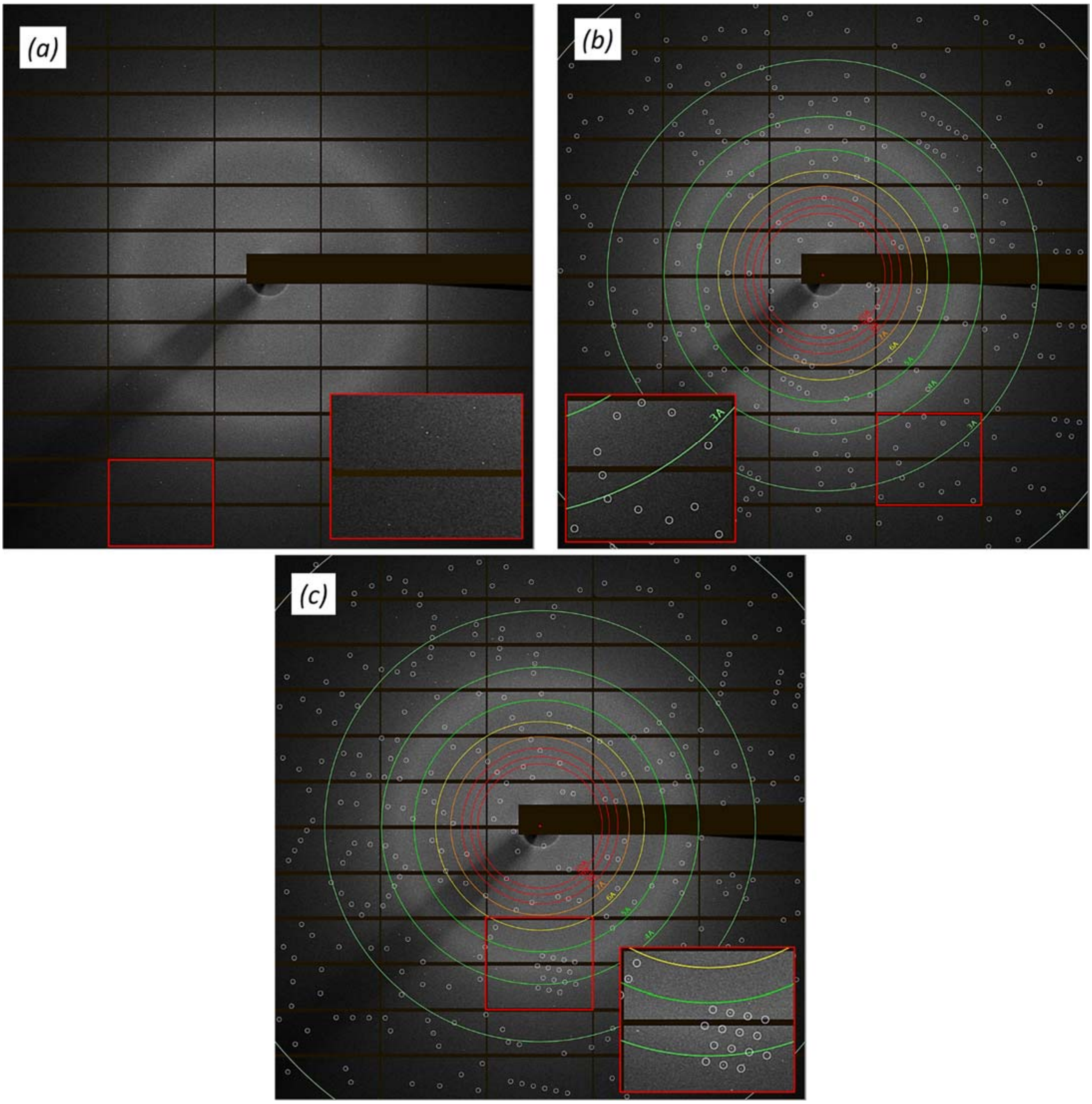

Figure S2. Diffraction from a couple of proteinase K crystals in different orientation. *(a)* Diffraction pattern showing Bragg peaks up to nearly 2 Å resolution. (*b*) Diffraction pattern in *(a)* showing the indexing solution of one of the two crystals. (*c*) Diffraction pattern in *(a)* showing the indexing solution of the other crystal.

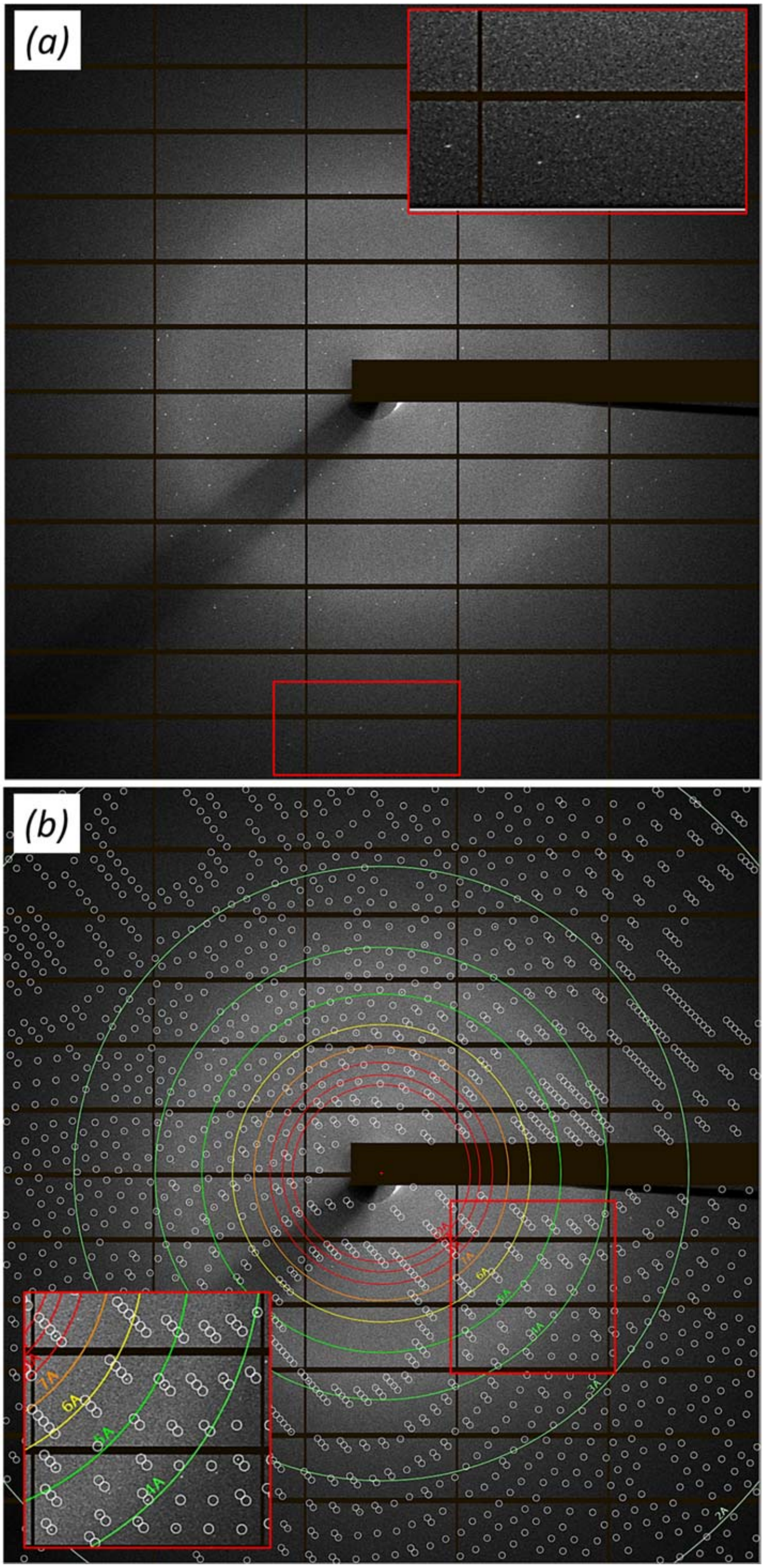

Figure S3. Diffraction pattern of a single micro-crystal of phycocyanin suspended in LCP. *(a)* Diffraction pattern showing Bragg peaks up to nearly 2 Å resolution. *(b)* Same diffraction pattern in *(a)* after indexing with resolution rings ON (showing the predicted spot locations).

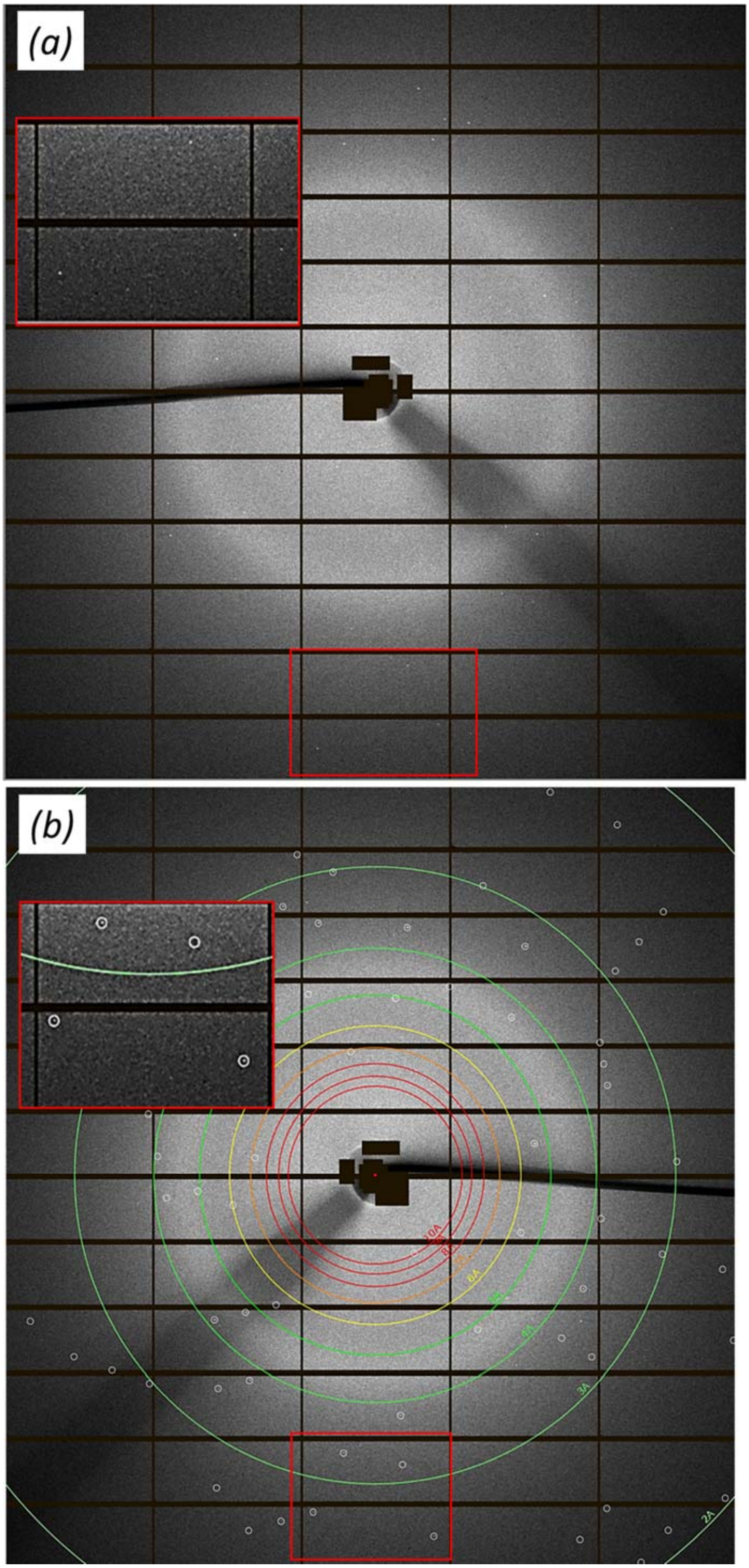

Figure S4. Diffraction pattern of a single micro-crystal of α-Spectrin-SH3 suspended in LCP. *(a)* Diffraction pattern showing Bragg peaks up to nearly 2 Å resolution. *(b)* Same diffraction pattern in *(a)* after indexing with resolution rings ON (showing the predicted spot locations).

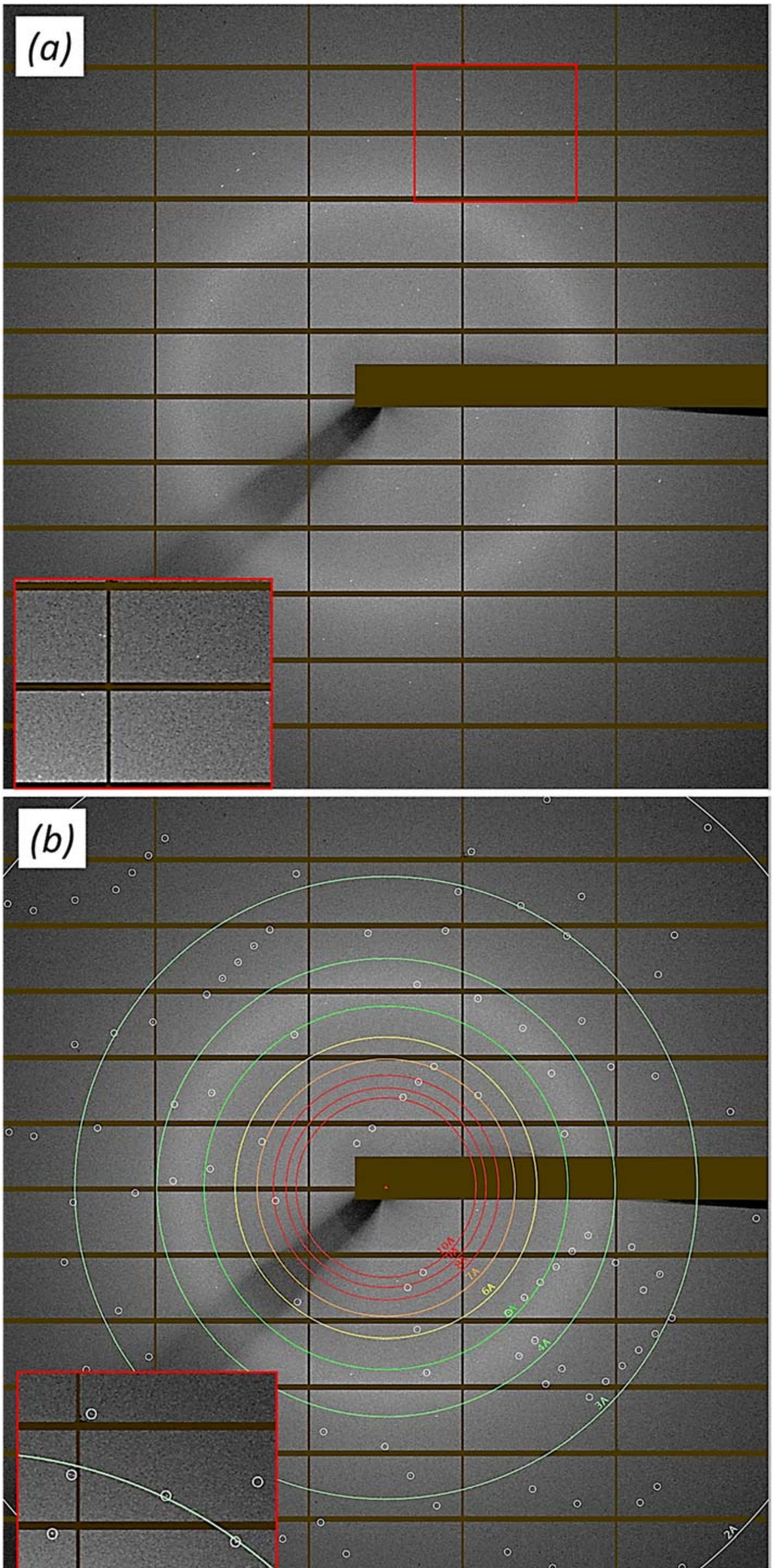

Figure S5. Diffraction pattern of a single micro-crystal of insulin suspended in LCP. *(a)* Diffraction pattern showing Bragg peaks up to nearly 2 Å resolution. *(b)* Same diffraction pattern in *(a)* after indexing with resolution rings ON (showing the predicted spot locations).

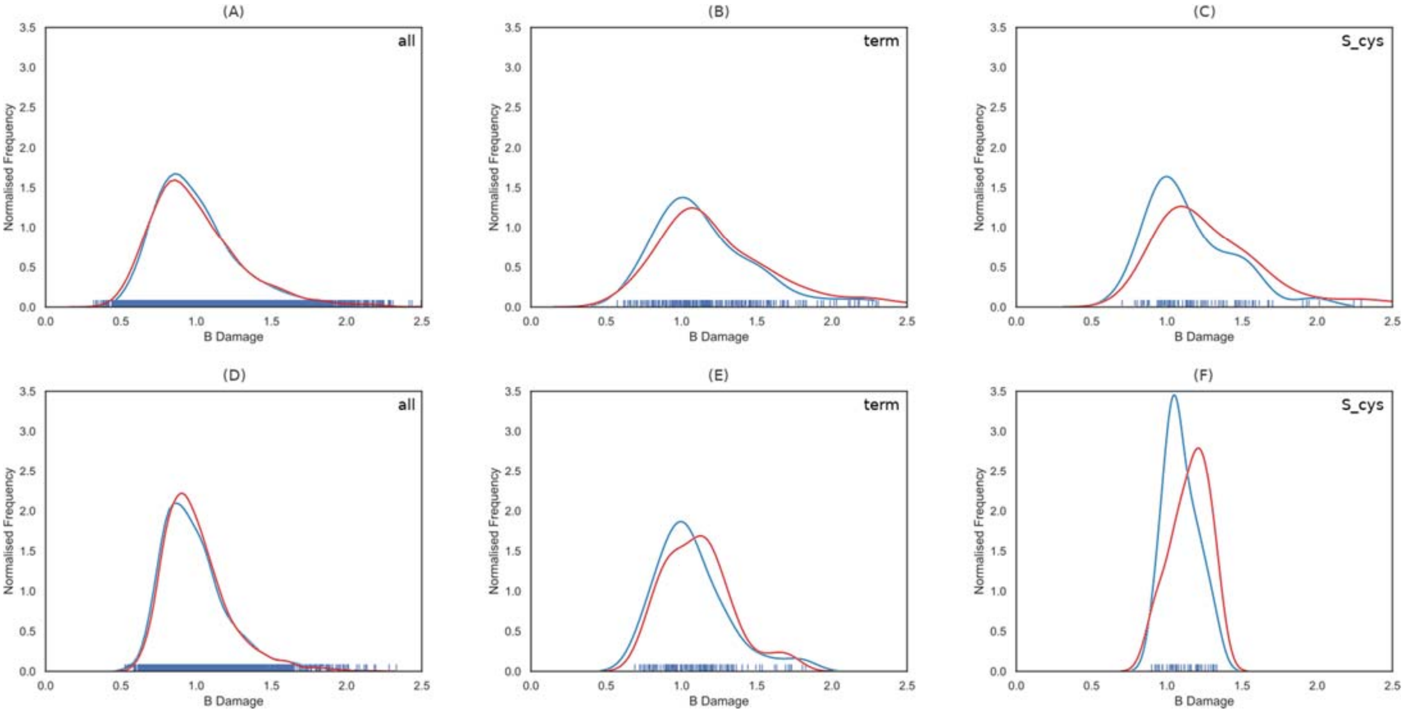

Figure S6. B_{Damage} distribution of two sets of reference structures. The distribution profiles have been calculated considering all protein atoms (*all;* A and D panels), the terminal GLU Oε, ASP Oδ and CYS Sγ atoms (*term*; B and E panels) or only CYS Sγ atoms (*S_cys*; C and F panels). In the *top* (Nanao et al. 2005) and *bottom* (Sutton et al. 2013) structure sets, the B_{Damage} distribution depicted in blue has been calculated using an ensemble, adding up all atom contributions, of the lowest radiation dose structures (*top*: 2BLR-thaumatin-, 2BLO-elastase-, 2BLP-RNAse-, 2BLV-trypsin-, 2BLX-lysozyme-, and 2BN3-insuline-, *bottom*: 4H8X, 4H8Y and 4H8Z lysozyme structures). In a similar manner, the B_{Damage} distribution depicted in red has been calculated using the highest radiation dose structures (*top*: 2BLU, 2BLQ, 2BLZ, 2BLW, 2BLY, and 2BN1, *bottom*: 4H8X, 4H8Y and 4H8Z)

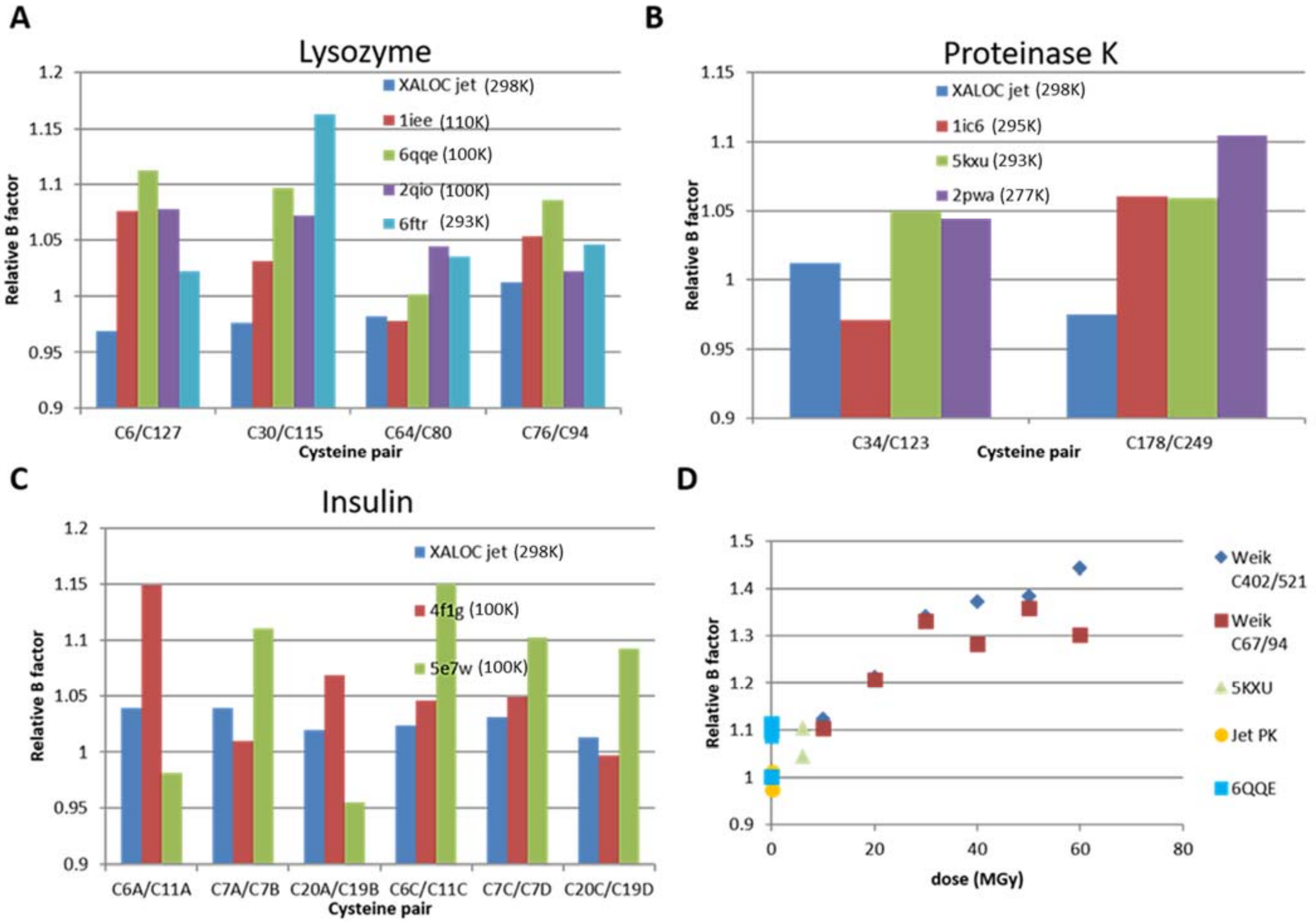

Figure S7. S_γ/C_β B factors of structures presented herein and those of relevant PDB structures for A) lysozyme, B) proteinase K and C) insulin. Panel D) shows the ratio of B-factor of the S_γ atom with respect to the C_β atom in the cysteines involved in the disulfide bonds of the selected PDB structures as a function of dose.

Supplemental Table 1: Data Collection statistics for reduced image, random subsets for Proteinase K

	**Proteinase K ALL**	**Proteinase K 100,000 patterns**	**Proteinase K 50,000 patterns**	**Proteinase K 10,000 patterns**	**Proteinase K 5,000 patterns**	**Proteinase K 4,000 patterns**	**Proteinase K 3,000 patterns**
Data collection time (h)	11	~ 5h 4min	~ 2h 32min	~ 30min	~ 15min	~ 12min	~ 9min
Sample consumption (µL)	19.4	8.95	4.48	0.90	0.45	0.36	0.27
No. of indexed patterns	216,645	98,775	49,005	9,654	4,758	3,800	2,840
Resolution range (Å)	48.4 – 1.9 (1.97 – 1.9)	48.4 – 1.9 (1.97 – 1.9)	48.4 – 1.9 (1.97 – 1.9)	48.4 – 1.9 (1.97 – 1.9)	48.4 – 1.9 (1.97 – 1.9)	48.4 – 1.9 (1.97 – 1.9)	48.4 – 1.9 (1.97 – 1.9)
Total No. of reflections	78,935,391 (640,218)	35,793,680	17,747,436 (144,617)	3,503,239 (28,413)	1,724,548 (14,079)	1,381,939 (11,298)	1,027,668 (8,263)
No. of unique reflections	21,010 (2,033)	21,010 (2,033)	21,010 (2,033)	21,010 (2,033)	21,010 (2,033)	21,010 (2,033)	21,010 (2,033)
Completeness (%)	100 (100)	100 (100)	100 (100)	100 (99.8)	99.7 (97.2)	99.4 (93.9)	98.5 (87.3)
Redundancy	3,757 (315)	1,704 (144)	845 (71)	167 (14)	82 (7)	66 (6)	50 (5)
⟨ I/σ(I)⟩	17.4 (0.6)	11.7 (0.4)	8.3 (0.3)	3.7 (0.1)	2.7 (0.1)	2.4 (0.1)	2.1 (0.0)
CC* (%)	99.96 (60.67)	99.92 (50.12)	99.85 (30.38)	99.13 (19.24)	98.43 (19.96)	98.09 (18.81)	97.51 (11.12)
R_{split}	4.6 (145.1)	6.7 (219.2)	9.6 (335.5)	22.1 (662.7)	30.1 (984.3)	32.8 (714.2)	37.5 (1113)
Wilson B factor (Å^2)	23.7	20.4	19.6	22.1	22.4	21.2	20.0

Supplemental Table 2: Data Collection statistics for reduced image, random subsets for Lysozyme.

	**Lysozyme ALL**	**Lysozyme 10,000 patterns**	**Lysozyme 5,000 patterns**	**Lysozyme 4,000 patterns**	**Lysozyme 3,000 patterns**
Data collection time (h)	2.6	~ 1h 6min	~ 32 min	~ 26 min	~ 20 min
Sample consumption (µL)	4.5	1.9	0.9	0.8	0.6
No. of indexed patterns	23,733	9,597	4,693	3,769	2,800
Resolution range (Å)	38.2 – 2.1 (2.17 – 2.1)	38.2 – 2.1 (2.17 – 2.1)	38.2 – 2.1 (2.17 – 2.1)	38.2 – 2.1 (2.17 – 2.1)	38.2 – 2.1 (2.17 – 2.1)
Total No. of reflections	5,089,514 (115,198)	2,057,288 (46,654)	1,011,093 (22,735)	808,905 (18,221)	599,881 (13,578)
No. of unique reflections	7,498 (716)	7,498 (716)	7,498 (716)	7,498 (716)	7,498 (716)
Completeness (%)	100 (100)	100 (100)	100 (100)	100 (100)	100 (100)
Redundancy	679 (161)	274 (65)	135 (32)	108 (25)	80 (19)
⟨ I/σ(I)⟩	9.8 (0.5)	6.3 (0.4)	4.4 (0.2)	4.1 (0.2)	3.5 (0.2)
CC* (%)	99.90 (58.55)	99.64 (44.78)	99.25 (17.76)	99.26 (17.42)	99.19 (17.80)
R_{split}	7.6 (157.9)	12.4 (265.1)	18.0 (426.2)	18.9 (430.7)	21.8 (502.9)
Wilson B factor (Å^2)	37.4	37.9	37.9	37.3	39.5

Supplemental Table 3: The median, B_{net} and variances of the B_{Damage} distributions from the several protein sets referred to in Figure 4 are reported.

	median	v_{all}	v_{term}	v_{S_cys}
Ins	0.99	6,5E-02	7,8E-03	5,7E-03
Phy	0.99	7,6E-03	7,3E-03	1,4E-02
Lyso	0.98	2,1E-02	8,8E-03	5,7E-03
PK	0.99	1,5E-02	1,1E-02	2,4E-03
SPC	0.98	2,8E-02	3,7E-02	- - - -
(middle)				
S1_4RLM	0.98	8,4E-03	3,7E-03	4,3E-03
S2_5UVJ	0.97	4,2E-02	8,1E-02	1,6E-02
X1_5DM9	0.97	2,7E-02	4,2E-02	9,0E-03
X2_6H0K	0.98	2,8E-02	3,4E-02	1,5E-02
X3_7BYO	0.97	3,9E-02	3,0E-02	1,5E-02
X4_6H0L	0.97	4,3E-02	3,3E-02	2,2E-02
RT1_1IEE	0.93	8,2E-02	7,1E-02	2,0E-02
RT2_6QQE	0.94	1,9E-01	4,2E-02	2,8E-02
(bottom)				
S1_5UVL	0.99	1,6E-02	2,4E-02	1,1E-02
S2_6FJS	0.95	7,3E-02	9,8E-02	7,7E-03
S3_6MH6	0.95	1,0E-01	1,1E-01	6,1E-02
